# ER-phagy requires the assembly of actin at sites of contact between the cortical ER and endocytic pits

**DOI:** 10.1073/pnas.2117554119

**Published:** 2022-01-31

**Authors:** Dongmei Liu, Muriel Mari, Xia Li, Fulvio Reggiori, Susan Ferro-Novick, Peter Novick

**Affiliations:** ^a^Department of Cellular and Molecular Medicine, University of California San Diego, La Jolla, CA 92093-0668;; ^b^Department of Biomedical Sciences of Cells and Systems, University of Groningen, University Medical Center Groningen, 9713 AV Groningen, The Netherlands

**Keywords:** autophagy, endoplasmic reticulum, actin

## Abstract

Fragments of the endoplasmic reticulum (ER) are selectively delivered to the lysosome (mammals) or vacuole (yeast) in response to starvation or the accumulation of misfolded proteins through an autophagic process known as ER-phagy. A screen of the *Saccharomyces cerevisiae* deletion library identified *end3Δ* as a candidate knockout strain that is defective in ER-phagy during starvation conditions, but not bulk autophagy. We find that loss of End3 and its stable binding partner Pan1, or inhibition of the Arp2/3 complex that is coupled by the End3-Pan1 complex to endocytic pits, blocks the association of the cortical ER autophagy receptor, Atg40, with the autophagosomal assembly scaffold protein Atg11. The membrane contact site module linking the rim of cortical ER sheets and endocytic pits, consisting of Scs2 or Scs22, Osh2 or Osh3, and Myo3 or Myo5, is also needed for ER-phagy. Both Atg40 and Scs2 are concentrated at the edges of ER sheets and can be cross-linked to each other. Our results are consistent with a model in which actin assembly at sites of contact between the cortical ER and endocytic pits contributes to ER sequestration into autophagosomes.

## Significance

Portions of the endoplasmic reticulum (ER) are degraded by autophagy (ER-phagy) in response to starvation or the accumulation of misfolded proteins. We show that ER-phagy requires assembly of actin at sites of contact between the edges of ER sheets and endocytic pits on the plasma membrane. Actin assembly may help to bring an element of the ER carrying the selective autophagy receptor Atg40 into the cell interior, where it associates with Atg11, a scaffold needed to recruit components for autophagosome assembly. Understanding the mechanism by which regions of the ER are selected for degradation and sequestered within autophagosomes may help in the development of novel approaches to treat diseases that result from the accumulation of misfolded proteins within the ER.

More than a third of the proteome translocates across the endoplasmic reticulum (ER) membrane and enters the ER lumen in an unfolded state (1). Even under normal conditions, a significant fraction of newly translocated polypeptides fails to properly fold (2). This can interfere with ER functions, including the folding and export of other proteins (3, 4). The degradation of compromised sections of the ER can occur by several different mechanisms. Here we focus on a type of selective autophagy known as macro-ER-phagy (hereafter ER-phagy) (5) that is induced by starvation or rapamycin treatment (5, 6). The ability to recycle components of the ER and to reduce the biosynthetic capacity of the ER to match a decrease in cellular demands is important for homeostasis. The mechanisms by which regions of the ER are recognized, severed from the ER network, and processed for degradation through ER-phagy are incompletely understood.

The ER is comprised of several distinct yet contiguous regions that together form one structure extending throughout the cell volume (7, 8). In many cell types, a polygonal network of smooth ER tubules is found at the cell periphery, while ribosome-studded ER sheets occupy a more central region of the cell. The outer nuclear membrane is also contiguous with the ER and shares many of its functions. Different degradation pathways have been implicated in the turnover of these different regions (9). ER-phagy involves the sequestration of ER segments by autophagosomes, which after closure deliver their contents into the lysosome (the vacuole in yeast) for degradation. This process utilizes ER-associated cargo receptors that allow selective recognition and incorporation into autophagosomes. Mammalian cells employ different ER-phagy receptors for the selective degradation of ER sheets and tubules (10). In the yeast *Saccharomyces cerevisiae*, Atg40 is the cargo receptor for ER-phagy of the cortical ER (cER), ER that is juxtaposed to the plasma membrane (PM), while Atg39 is the cargo receptor for ER-phagy of the nuclear envelope (11). All autophagy receptors bind to Atg8 (yeast), LC3, or GABARAP proteins (mammals) (10, 12). Here we have focused on Atg40-dependent cER-phagy. This pathway can be induced by the TOR inhibitor rapamycin, starvation, or the accumulation of aggregation-prone proteins (11, 13). Atg40 shares similarities with two different mammalian ER-phagy receptors, the FAM134 protein family and RTN3L (11, 14, 15). Atg40 has the same domain structure as the ER sheets receptor FAM134B (11, 14), yet it primarily localizes to ER tubules like RTN3L (11, 15, 16). Atg40, FAM134 proteins, and RTN3L all contain reticulon homology domains (11, 14, 15).

Other than ER-phagy receptors, only a few additional components have been implicated in recognizing, severing and sequestering fragments of the ER for degradation. Recent studies have demonstrated a noncanonical role for the COPII vesicle coat subunit, Lst1 (SEC24C in mammals), in ER-phagy. Lst1, which forms a complex with Sec23, works in conjunction with Atg40 to select ER subdomains for degradation (13). Additionally, Lnp1, a protein that promotes remodeling of the cER network by stabilizing newly formed three-way junctions (17, 18), is required for cER-phagy (19). The roles of Lst1 and Lnp1 in ER-phagy appear to be evolutionarily conserved (20). Inhibition of actin polymerization with the drug latrunculin A (LatA) blocks ER-phagy (6) as well as other selective autophagy pathways in yeast (21). LatA also prevents the association of Atg40 with the autophagosome assembly scaffold protein, Atg11 (19). This step precedes the sequestration of the ER by autophagosomes at the phagophore assembly site (PAS), perivacuolar sites where autophagosomes form (22). These observations suggest that actin-dependent ER remodeling and/or transport may be needed to bring elements of the cER carrying Atg40 to the PAS.

In an effort to identify additional components of the ER-phagy machinery, we performed a systematic screen for mutants defective in the selective autophagic turnover of the cER. This screen led to the identification of a number of candidates, including the *VPS13* gene (23). *VPS13* encodes a protein that mediates phospholipid transfer at interorganelle contact sites (23, 24). In the absence of Vps13, the late endosome enlarges. Interestingly, the loss of Vps13 does not disrupt the association of Atg40 with Atg11, but instead blocks the subsequent sequestration of ER into autophagosomes (23). Understanding the components and mechanisms involved in ER-phagy could also reveal new approaches to treat human disease conditions in which ER function is impaired. A number of the components involved in ER-phagy have been implicated in several pathologies, including different forms of hereditary spastic paraplegias and Parkinson’s disease (25). Here, we have pursued another candidate from our initial screen, and this has led us to investigate the role of actin assembly components in cER-phagy.

## Results

### The Identification of End3 in a Screen for ER-phagy Mutants.

In a recent genome-wide screen of the yeast deletion library, we identified >20 mutants that reduce ER-phagy, but not bulk autophagy (23). In that study, cells were treated for 16 h with rapamycin, and the vacuolar delivery of two different GFP-tagged ER membrane proteins, Rtn1 and Sec61, was monitored (23). Rtn1 marks ER tubules and the edges of ER sheets (8, 26), while Sec61 localizes to both the cER and nuclear ER (17). Among the genes identified was *END3*. Here we focus on the role of End3 and associated proteins in cER-phagy.

Since the screen was performed using the deletion library constructed in the BY strain background, we first deleted *END3* in our NY strain background (both strains originally derived from the S288c background) to confirm the initial findings. The *end3Δ* strain that we constructed showed a much more severe effect of rapamycin on cell morphology than the strain from the deletion library, with many dead cells 16 h after rapamycin addition (*SI Appendix*, Fig. S1*A*). We also deleted *END3* in the parental BY strain. The resulting *end3Δ* strain responded similarly to the one made in our background (*SI Appendix*, Fig. S1*A*), suggesting that the relative health of the deletion library strain does not reflect a background-specific difference, but is probably due to a suppressor mutation acquired by the *end3Δ* strain while passaging the library collection. In the absence of rapamycin, the newly constructed *end3Δ* strains were temperature sensitive for growth, while the strain retrieved from the library was temperature resistant, supporting this interpretation (*SI Appendix*, Fig. S1*B*).

### Induced Degradation of End3 and Pan1 Inhibits the Colocalization of Atg11 and Atg40.

**Fig. 1.**
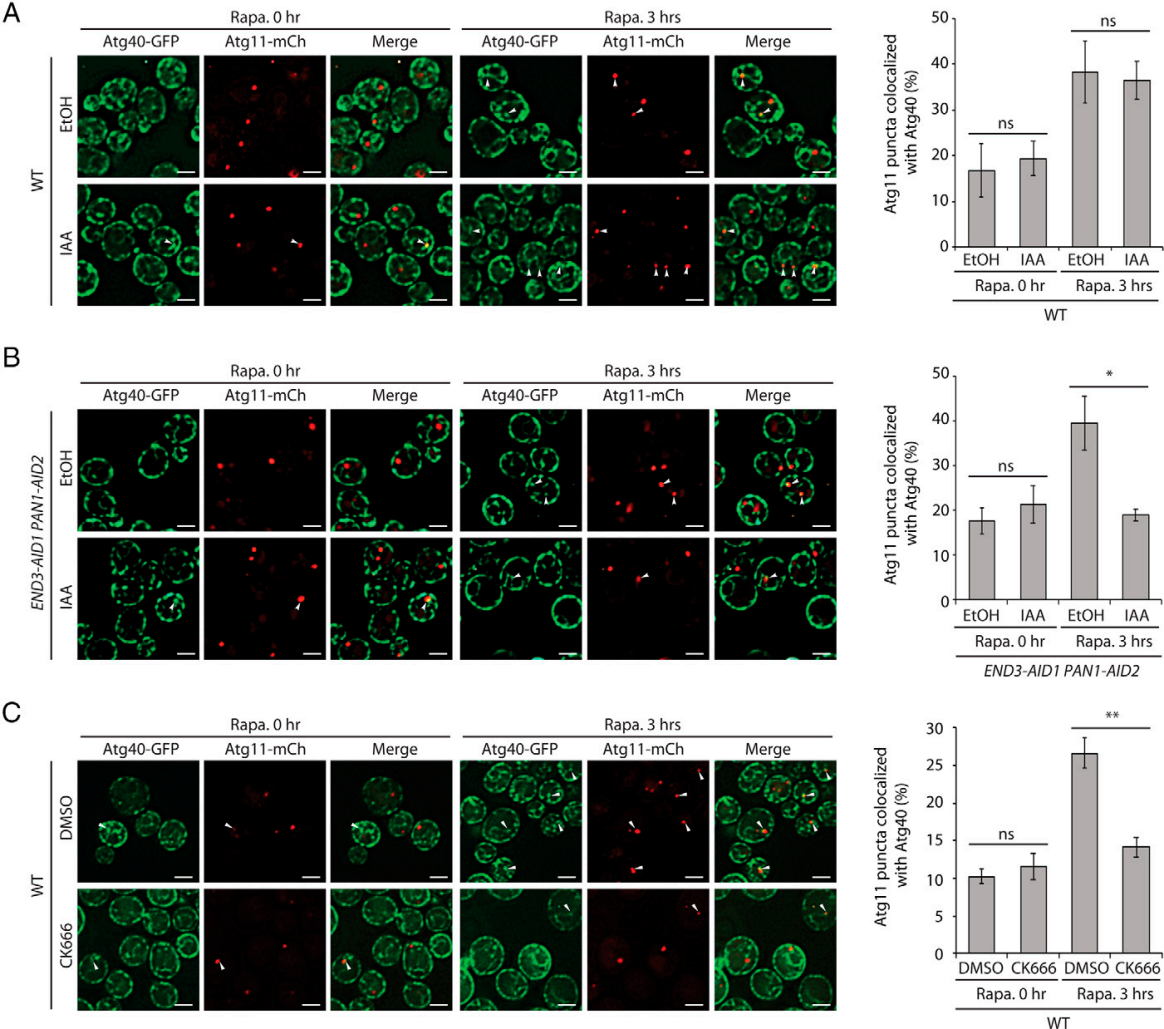
Induced degradation of End3 and Pan1 or inhibition of Arp2/3 with CK-666 inhibits the colocalization of Atg11 and Atg40. (*A*) *Left*: Fluorescence images of yeast cells expressing both Atg40-3xGFP and Atg11-2xmCherry before and after a 3-h treatment with rapamycin (Rapa.). Cells were pretreated with either EtOH or IAA for 90 min before the addition of rapamycin. The arrowheads point to Atg11 puncta that colocalize with Atg40 puncta. (Scale bars, 2 μm.) *Right*: The percentage of Atg11 puncta that colocalizes with Atg40 was quantified. Error bars represent SDs, *n* = 3 independent experiments. ns, nonsignificant (*P* ≥ 0.05); *P < 0.05; **P < 0.01. Student’s *t* test. (*B*) *Left*: Fluorescence images of yeast cells expressing End3-AID1, Pan1-AID2, Atg40-3xGFP, and Atg11-2xmCherry before and after 3-h rapamycin treatment. Cells were pretreated with either EtOH or IAA for 90 min before the addition of rapamycin. The arrowheads point to Atg11 puncta that colocalize with Atg40 puncta. (Scale bars, 2 μm.) *Right*: The percentage of Atg11 puncta that colocalizes with Atg40 was quantified. Error bars represent SDs, *n* = 3 independent experiments. ns, nonsignificant (*P* ≥ 0.05); *P < 0.05; **P < 0.01. Student’s *t* test. (*C*) *Left*: Fluorescence images of yeast cells expressing Atg40-3xGFP and Atg11-2xmCherry before and after 3-h rapamycin treatment. Cells were pretreated with either DMSO or CK-666 for 90 min before the addition of rapamycin. The arrowheads point to Atg11 puncta that colocalize with Atg40 puncta. (Scale bars, 2 μm.) *Right*: The percentage of Atg11 puncta that colocalizes with Atg40 was quantified. Error bars represent SDs, *n* = 3 independent experiments. ns, nonsignificant (*P* ≥ 0.05); *P < 0.05; **P < 0.01. Student’s *t* test.

End3 is an Epsin15 homology (EH) domain–containing protein. In vivo it forms a stable complex with Pan1, another EH domain–containing protein. The Pan1-End3 complex is joined by Sla1 to regulate actin cytoskeleton organization and endocytosis (27–29). By interacting with multiple binding partners, including type I myosins, Ent1, Ent2, Yap1801, and Yap1802, the End3-Pan1-Sla1 complex acts at discrete locations on the PM to coordinate endocytic site initiation with the localized assembly of actin (29). Since the *end3Δ* strain is too sick to be analyzed after extended rapamycin treatment (*SI Appendix*, Fig. S1*A*) and Pan1 is essential for growth, we rapidly depleted cells of both End3 and Pan1 using the auxin-inducible degron (AID) system (30, 31). The strain simultaneously expressing AID-tagged End3 and Pan1 used for this study has previously been described by the Drubin laboratory (29). Upon the addition of auxin, both proteins are depleted within 1 h (29). While cell death prevented the analysis of the delivery and degradation of ER membrane proteins following rapamycin addition for 16 h, we were able to assess the association of Atg11 with Atg40 puncta on elements of the ER after 3 h of rapamycin treatment. These sites of colocalization could represent locations where ER fragmentation and sequestration into autophagosomes take place. After inducing the degradation of End3 and Pan1 by addition of the auxin family hormone IAA for 90 min (30), rapamycin was added. The colocalization of Atg40-3xGFP puncta with Atg11-2xmCherry was assessed before and after 3 h of rapamycin treatment. Before rapamycin addition, cells that were mock-treated with ethanol (EtOH), or treated with IAA, showed a similar basal level of Atg40-Atg11 colocalization (Fig. 1*B*). After inducing ER-phagy with rapamycin, we observed an increase in colocalization in the mock-treated but not the IAA-treated cells (Fig. 1*B*). This inhibition was not observed in wild-type (WT) cells that were treated with IAA (Fig. 1*A*), which ruled out a nonspecific effect of this drug. This observation suggests that the function of the Pan1-End3 complex is important for the association of Atg40 puncta with Atg11 puncta. A similar reduction in Atg40-Atg11 colocalization was observed when actin polymerization was blocked with LatA (19). Therefore, since the Pan1-End3 complex is known to coordinate endocytic site initiation with the localized assembly of actin (29), the inhibition in the colocalization of Atg40 with Atg11 could be due to the lack of actin assembly at endocytic sites.

### Inhibition of Arp2/3 with CK-666 Inhibits Atg40-Atg11 Colocalization.

Actin assembly can be catalyzed by either the Arp2/3 complex at endocytic sites or by the formins at bud tips or bud necks. The Arp2/3 complex is composed of seven subunits: Arp2, Arp3, Arc15, Arc18, Arc19, Arc35, and Arc40 (32). Upon activation by WASP protein family members, the Arp2/3 complex initiates the formation of a new actin filament that emerges from an existing filament in a y-branch configuration. CK-666 is a reversible small molecule inhibitor of the Arp2/3 complex (33). To further pursue the role of endocytosis and coupled actin assembly in ER-phagy, we treated WT cells with either dimethyl sulfoxide (DMSO) or CK-666. Because of extensive cell death, it was not possible to analyze ER degradation after 16 h of concurrent rapamycin and CK-666 treatment. Therefore, we analyzed Atg40-Atg11 colocalization after 3 h. Before rapamycin induction, both treatments showed a basal level of Atg40-Atg11 colocalization (Fig. 1*C*). After a 3-h incubation with rapamycin, cells mock-treated with DMSO displayed a 2.7-fold increase in colocalization that was strongly inhibited by CK-666 (Fig. 1*C*). The observation that either inhibiting actin assembly or uncoupling endocytic site initiation from actin assembly disrupted Atg40-Atg11 colocalization supports a role for actin assembly at endocytic sites in cER-phagy.

### Proteins Linking the cER to Endocytic Sites Are Important for ER-phagy.

Endocytic invaginations transiently associate with the rim of cER sheets in yeast (34, 35). This interaction is mediated by three proteins, each represented by a pair of homologs: Scs2 or Scs22, Osh2 or Osh3, and Myo3 or Myo5 (35). For brevity, we will refer to these pairs as Scs2/22, Osh2/3, and Myo3/5. Scs2/22 are VAMP-associated proteins (VAP), and like other members of the VAP family, they are integral ER membrane proteins (36). Scs2/22 play largely redundant roles in the regulation of phospholipid metabolism, although Scs2 is the more dominant player (37, 38). *OSH2/3* are two out of the seven yeast *OSH* genes encoding oxysterol binding protein–related proteins (ORPs). They are recruited onto the cytoplasmic surface of the ER through interactions with Scs2/22 (39), yet can also interact with the type I myosins, Myo3/5 (35). In contrast to the type V myosins, which move along polarized actin cables toward sites of cell surface growth, Myo3/5 localize to cortical actin patches at endocytic sites to facilitate endocytosis (40, 41).

**Fig. 2.**
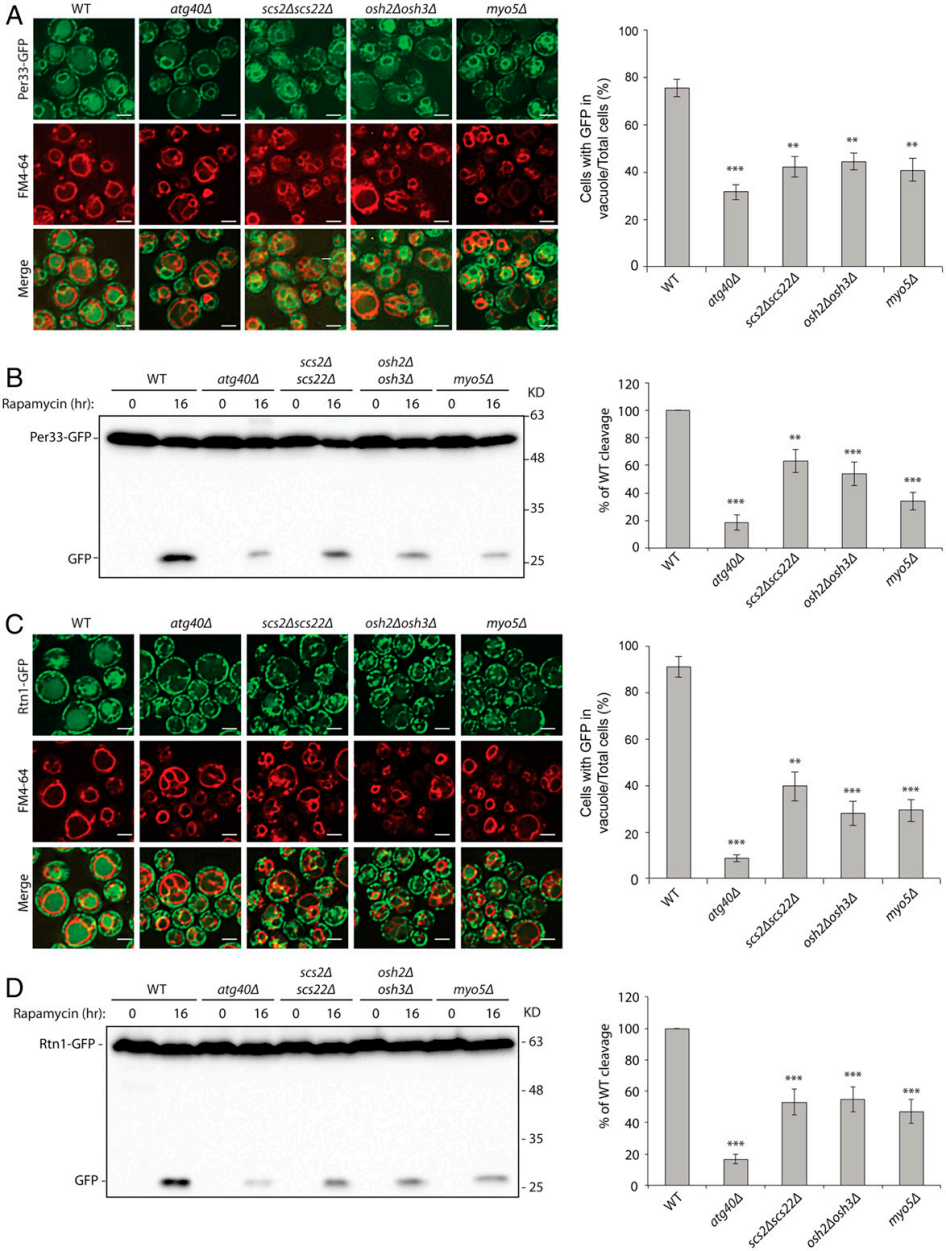
Proteins linking the cER to endocytic sites are important for ER-phagy. (*A*) Scs2/Scs22, Osh2/Osh3, and Myo5 are required for the delivery of Per33 into the vacuole. *Left*: Fluorescence images of cells expressing Per33-GFP. Cells were treated with rapamycin for 16 h, and vacuoles were stained with fn4-64. *Right*: The percentage of cells with GFP in the vacuole was quantified. Error bars represent SD, *n* = 3 independent experiments. **P < 0.01; ***P < 0.001. Student’s *t* test. (Scale bars, 2 μm.) (*B*) *Left*: Western blot of Per33-GFP cleavage after cells were treated with rapamycin for 16 h. *Right*: Percentage of free GFP divided by the total GFP amount was quantified. WT was set as 100%. All mutants were normalized to the WT control. Error bars represent SD, *n* = 3 independent experiments. **P < 0.01; ***P < 0.001. Student’s *t* test. (*C*) Scs2/Scs22, Osh2/Osh3, and Myo5 are required for the delivery of Rtn1 into the vacuole. *Left*: Fluorescence images of cells expressing Rtn1-GFP. Cells were treated with rapamycin for 16 h, and vacuoles were stained with fn4-64. *Right*: The percentage of cells with GFP in the vacuole was quantified. Error bars represent SD, *n* = 3 independent experiments. **P < 0.01; ***P < 0.001. Student’s *t* test. (Scale bars, 2 μm.) (*D*) *Left*: Western blot analysis of Rtn1-GFP cleavage after cells were treated with rapamycin for 16 h. *Right*: Percentage of free GFP divided by the total GFP amount was quantified. WT was set as 100%. All mutants were normalized to the WT control. Error bars represent SD, *n* = 3 independent experiments. ***P < 0.001. Student’s *t* test.

Given the requirement for the Pan1-End3 and Arp2/3 complexes in cER-phagy, we explored the possibility that the components that link the cER to endocytic sites might also play a role. We assayed the *scs2Δ scs22Δ*, *osh2Δ osh3Δ*, and *myo5Δ* mutants for defects in cER-phagy. The *myo5Δ* strain was chosen instead of the double *myo3Δ myo5Δ* knockout because the double mutant became too sick for analysis after autophagy induction with rapamycin. Two activities were assayed: the delivery of ER marker proteins into the vacuole as measured by fluorescence microscopy and the vacuolar degradation of these marker proteins as measured by Western blot analysis. Three ER marker proteins were followed: Per33, Rtn1, and Hmg1. While Per33 is a transmembrane protein that resides in both the cER and the nuclear envelope (42), Rtn1 predominantly localizes in the cER (17), and Hmg1 is only present in the nuclear envelope (43). All three mutants, *scs2Δ scs22Δ*, *osh2Δ osh3Δ*, and *myo5Δ*, showed similar levels of reduction in the delivery of the tested ER marker proteins to the vacuole by fluorescence microscopy (Fig. 2 *A* and *C* and *SI Appendix*, Fig. S2*A*). These results were confirmed when we quantified the extent to which Per33-GFP, Rtn1-GFP, and Hmg1-GFP were cleaved in the vacuole to yield free GFP (Fig. 2 *B* and *D* and *SI Appendix*, Fig. S2*B*). Shorter exposures of the blots shown in Fig. 2 *B* and *D* are presented in *SI Appendix*, Fig. S3.

In addition to ER-phagy, rapamycin also induces nonselective bulk autophagy, which, like ER-phagy, requires autophagosome formation. To determine if the defects we observed could be due to a defect in autophagosome assembly, we performed two different bulk autophagy assays. When bulk autophagy is induced, cytosolic GFP-Atg8 is lipidated and incorporated into the autophagosomal membrane. Following fusion of the autophagosome with the vacuole, the GFP-Atg8 pool in the interior of autophagosomes is cleaved, releasing free GFP into the lumen (44). This cleavage assay indicated that none of the three mutants, *scs2Δ scs22Δ*, *osh2Δ osh3Δ*, or *myo5Δ*, displayed a reduced level of GFP-Atg8 cleavage; rather, *osh2Δ osh3Δ* and *myo5Δ* showed a slight increase relative to WT (*SI Appendix*, Fig. S4*A*). To confirm this result, we performed a second bulk autophagy assay that monitors the enzymatic activity of N-terminally truncated Pho8 (Pho8Δ60) (45). *PHO8* encodes a vacuolar alkaline phosphatase with a single membrane-spanning domain at its N terminus. Removing the 60 N-terminal amino acids yields a soluble cytosolic protein that is inactive due to an inhibitory domain. When bulk autophagy is induced by starvation or rapamycin treatment, a fraction of Pho8Δ60 is transported into the vacuole, and the inhibitory domain is cleaved by resident proteases. As a result, the phosphatase activity of Pho8Δ60 reflects the level of bulk autophagy (45). The Pho8Δ60 assay revealed that none of the three mutants has a significant reduction in bulk autophagy relative to the WT (*SI Appendix*, Fig. S4*B*). In contrast, the *atg1Δ* control strain, which is defective in autophagosome assembly, did not display bulk autophagy, as expected. Taken together, these two assays confirmed that the ER-phagy defects we observed in *scs2Δ scs22Δ*, *osh2Δ osh3Δ*, and *myo5Δ* mutants are not due to a general defect in autophagosome formation. We also confirmed that the induced simultaneous depletion of both End3 and Pan1 (*SI Appendix*, Fig. S4*C*) does not affect the delivery of Pho8Δ60 to the vacuole.

**Fig. 3.**
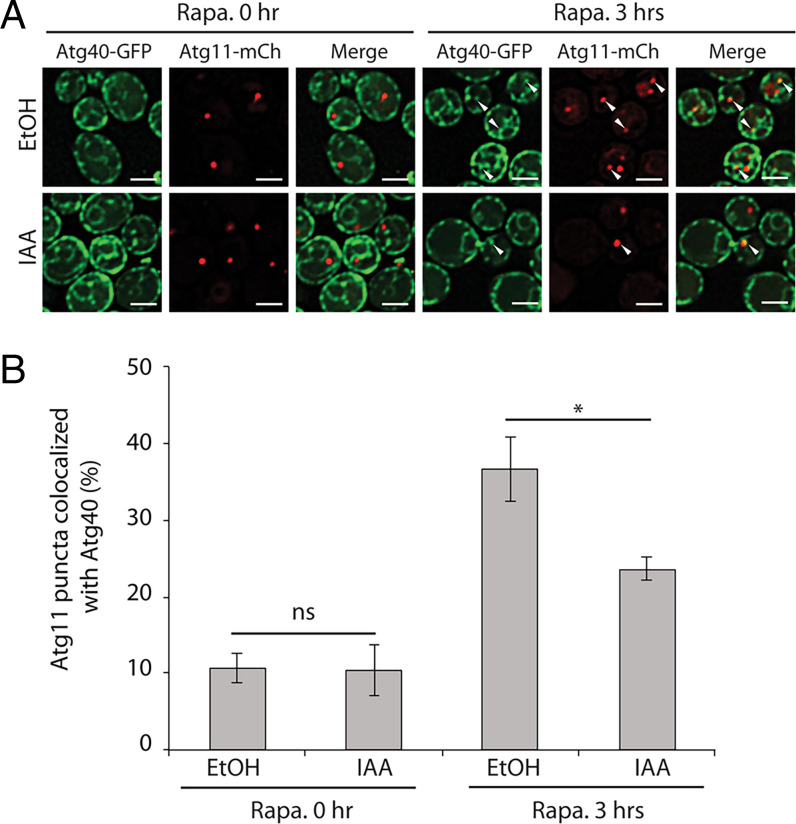
Induced degradation of Myo3 and Myo5 inhibits the colocalization of Atg11 and Atg40. (*A*) Fluorescence images of yeast cells expressing Myo3-AID1, Myo5-AID2, Atg40-3xGFP, and Atg11-2xmCherry before and after 3-h rapamycin (Rapa.) treatment. Cells were pretreated with either EtOH or IAA for 90 min before the addition of rapamycin. The arrowheads point to Atg11 puncta that colocalize with Atg40 puncta. (Scale bars, 2 μm.) (*B*) The percentage of Atg11 puncta that colocalize with Atg40 was quantified. Error bars represent SD, *n* = 3 independent experiments. ns, nonsignificant (*P* ≥ 0.05); *P < 0.05. Student’s *t* test.

To establish the stage at which the cER-endocytic bridge acts in ER-phagy, we induced the simultaneous depletion of Myo3 and Myo5 using the AID system and examined the colocalization of Atg40 puncta and Atg11 puncta. To confirm the efficacy of the induced depletion of Myo3/5, we measured the endocytic uptake of fn4-64 from the PM to the vacuole membrane (*SI Appendix*, Fig. S5*A*). This demonstrated a dramatic reduction in endocytosis, consistent with the loss of Myo3/5 function. As shown in Fig. 3 *A* and *B*, rapamycin induced about a threefold increase in colocalization. This increased colocalization was reduced by the addition of IAA (Fig. 3 *A* and *B*). These results are consistent with our results showing that depletion of the Pan1-End3 complex or inhibition of the Arp2/3 complex leads to reduced colocalization of Atg40 puncta with Atg11. Together, these data indicate that proteins serving to link endocytic site initiation and actin assembly (Pan1-End3), catalyze actin patch assembly (Arp2/3 complex), or connect the endocytic pit with the cER (Myo3/5) display similar cER-phagy defects when depleted. Thus, the bridge between the cER and endocytic sites might help to present cER domains marked by Atg40 to Atg11 puncta and thereby promote the formation of ER-containing autophagosomes.

In principle, the block in ER-phagy observed in mutants affecting components that act at endocytic sites could be due to either their effect on actin assembly at these sites or the resulting block in endocytosis. To resolve these possibilities, we measured ER-phagy in strains deleted for *RVS167*. This gene encodes an amphiphysin homolog that is essential for the membrane scission event leading to the formation of free endocytic vesicles (46, 47). This activity is required after the assembly of actin by the Arp2/3 complex that promotes invagination of the endocytic pits (48). We observed no decrease in the cleavage of Rtn1-GFP following rapamycin treatment of *rvs167Δ* cells relative to the WT control (*SI Appendix*, Fig. S5 *C* and *D*). An fn4-64 uptake assay confirmed the block in endocytosis in the *rvs167Δ* cells (*SI Appendix*, Fig. S5*B*). These results indicate that a block in endocytosis is not sufficient to block cER-phagy.

### Scs2/Scs22 and Osh2/Osh3 Promote Formation of ER-Containing Autophagosomes.

**Fig. 4.**
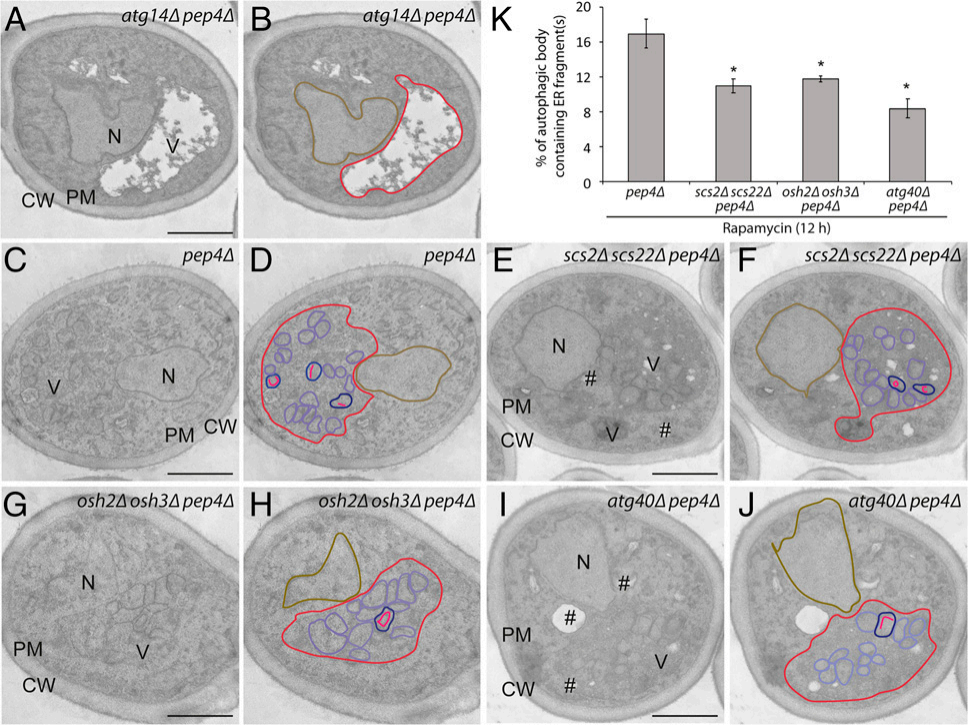
Scs2/Scs22 and Osh2/Osh3 are required for the sequestration of ER fragments into autophagic bodies (ABs). Cells were treated with rapamycin for 12 h and then fixed with permanganate, embedded in Spurr’s resin, and processed for electron microscopy. (*A* and *B*) Images of the *atg14Δ pep4Δ* strain. (*C* and *D*) Images of the *pep4Δ* strain. (*E* and *F*) Images of the *scs2Δ scs22Δ pep4Δ* cells. (*G* and *H*) Images of the *osh2Δ osh3Δ pep4Δ* strain. (*I* and *J*) Images of the *atg40Δ pep4Δ* cells. The location of vacuole (V), nucleus (N), cell wall (CW), PM, and lipid droplet (#) are marked in *A*, *C*, *E*, *G*, and *I*. Vacuole (red), nucleus (brown), AB (purple), AB containing ER fragments (blue), and ER fragments inside AB (pink) are outlined in *B*, *D*, *F*, *H*, and *J*. (*K*) Quantitation of the percentage of ABs containing ER. *P < 0.05. Student’s *t* test. (Scale bars, 1 µm.)

Next we performed thin section transmission electron microscopy to determine whether the link between the cER and endocytic pits is important for the packaging of ER into autophagosomes (13, 23). This analysis was performed with the *scs2Δ scs22Δ* and the *osh2Δ osh3Δ* mutants but not *myo3Δ myo5Δ* cells because this double mutant cannot tolerate the extended rapamycin treatment necessary for this study (Fig. 4). We quantified the percentage of autophagic bodies (ABs) that contain ER fragments, visible as linear segments (Fig. 4*K*) in a *pep4Δ* background. The Pep4 protease is needed to activate vacuolar hydrolases, and in its absence, ABs accumulate within the vacuolar lumen following rapamycin treatment (Fig. 4 *C* and *D*) (13, 23). This analysis revealed that both the *scs2Δ scs22Δ* and the *osh2Δ osh3Δ* strains exhibited a significantly reduced percentage of ABs containing ER fragments (Fig. 4 *E*–*H* and *K*). The reduction was somewhat less severe than what was observed in the *atg40Δ* strain (Fig. 4 *I*–*K*). No ABs were found in the *atg14Δ* mutant control (Fig. 4 *A* and *B*), which is known to block autophagosome formation (49). Thus, the link between the cER and endocytic pits is required prior to the packaging of ER fragments into autophagosomes.

### Scs2 Interacts with Atg40 In Vivo.

**Fig. 5.**
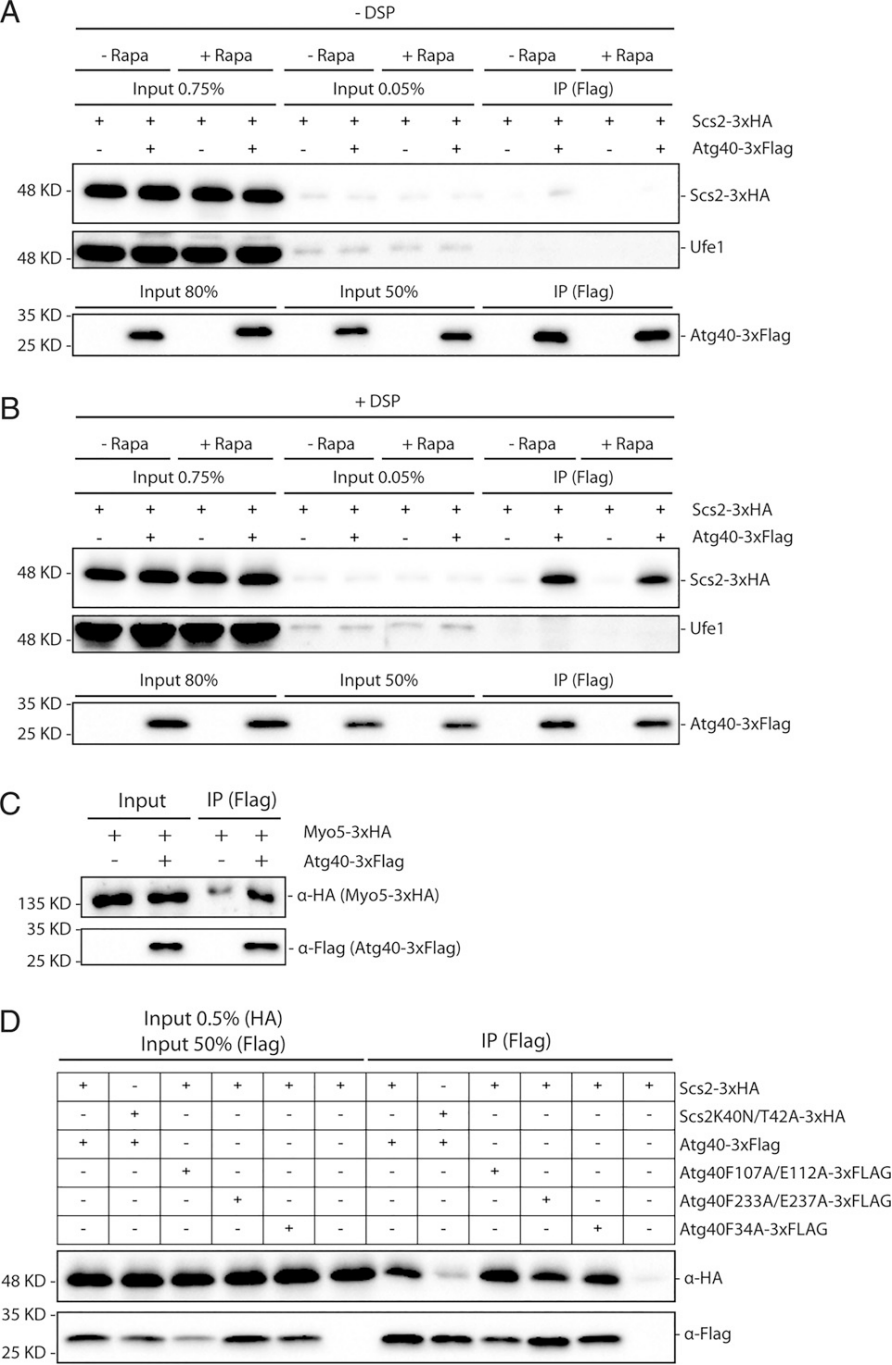
(*A*–*C*) Scs2 and Myo5 interact with Atg40 in vivo. (*A*) Atg40-3xFlag was immunoprecipitated from cell lysates, with or without prior rapamycin (Rapa) treatment. The immunoprecipitates (IPs) were blotted with anti-HA antibodies to detect Scs2-3xHA, Ufe1 rabbit antiserum to detect Ufe1, and anti-Flag antibodies to detect Atg40-3xFlag. Cross-linking reagent DSP was not used. (*B*) Same as described in *A* except the cross-linking reagent DSP was used prior to cell lysis. (*C*) Same protocol used as in *B* except cells expressed Atg40-3xFlag and Myo5-3xHA. Input represents 0.1% of the total for Myo5-3xHA and 50% for Atg40-3xFlag. (*D*) The FFAT domain–binding region of Scs2 is important for its interaction with Atg40 in vivo. Atg40-3xFlag (or its mutants) was immunoprecipitated from cell lysates treated with rapamycin for 3 h. The precipitates were blotted with anti-HA antibodies to detect Scs2-3xHA (or its mutant), and anti-Flag antibodies to detect Atg40-3xFlag (or its mutants). Cross-linking reagent DSP was used.

Since Scs2 and Atg40 are both integral ER membrane proteins, we determined whether they physically interact with each other. To explore a possible interaction, we performed a coprecipitation assay using Scs2-3xHA and Atg40-3xFlag. Atg40-3xFlag was overexpressed from the *CUP1* promoter, since its endogenous expression levels are too low to be examined, while Scs2-3xHA was expressed endogenously. We immunoprecipitated Atg40-3xFlag with anti-Flag antibody–conjugated beads and then used anti-HA antibody to detect the associated Scs2-3xHA. We observed little to no interaction between Atg40 and Scs2 in lysates of cells either untreated or treated with rapamycin for 3 h (Fig. 5*A*). However, when we used the cross-linking reagent dithiobissuccinimidyl propionate (DSP) prior to lysis, both rapamycin-treated and rapamycin-untreated cell lysates showed a robust interaction between Atg40 and Scs2 (∼0.75% of input, Fig. 5*B*), suggesting that the interaction might be transient, and not dependent on rapamycin treatment. This interaction appears to be specific since Atg40 did not exhibit a detectable interaction with Ufe1, another ER membrane protein (Fig. 5*B*). Additionally, using the same methodology, we observed an interaction above background between Atg40-3xFlag and Myo5-3xHA. Approximately 0.1% of the Myo5-3xHA coprecipitated with Atg40-3xFlag (Fig. 5*C*). Since Scs2 is located on both the cER and nuclear envelope (37), a coprecipitation assay was also performed with the nucleophagy receptor Atg39 (11). Interestingly, a robust interaction between these two proteins was also observed (*SI Appendix*, Fig. S6).

Scs2 is known to interact with proteins containing a FFAT motif. Several important sites have been identified within Scs2 that are required for its interaction with FFAT motif–containing proteins, including K40, T42, and K120. The Scs2^K40A^ mutant exhibits a strong defect both in ER localization of FFAT motif–containing proteins and on inositol dependence (37). Using the algorithm provided by Murphy et al. (50), we did not identify strong FFAT or FFAT-like motifs within Atg40, but found a few low-scoring sites, including F34, F107, E112, F233, and E237. Atg40^F107A/E112A^, Atg40^F233A/E237A^, and Atg40^F34A^ variants, however, all failed to disrupt the interaction with Scs2 (Fig. 5*D*). In contrast, the Scs2^K40N/T42A^ mutant displayed a dramatically reduced interaction with Atg40 (Fig. 5*D*). Taken together, these data suggest that Scs2 interacts with Atg40 either directly, through a novel, unidentified FFAT-binding motif, or more likely, indirectly through a protein(s) that contains a FFAT motif. Interestingly, two known interactors of Scs2 that have a FFAT motif are Osh2 and Osh3 (39). These two proteins are also part of a bridge that connects the cER with endocytic sites, and therefore they represent good candidates to mediate the Scs2–Atg40 interaction. To test this hypothesis, we repeated the Scs2-Atg40 binding assay with lysates from *osh2Δ*, *osh3Δ*, and *osh2Δ osh3Δ* cells, yet the Scs2–Atg40 interaction was not affected in any of the mutants tested (*SI Appendix*, Fig. S7*A*), suggesting that Osh2 and Osh3 do not mediate this interaction. Vps13 is another protein implicated in ER-phagy that contains a FFAT motif (23); however, deletion of *VPS13* also failed to disrupt the Scs2–Atg40 interaction (*SI Appendix*, Fig. S7*B*).

Since the FFAT motif–binding domain of Scs2 is critical for its association with Atg40, we further tested whether this feature is also critical for cER-phagy. We performed cleavage assays using three different ER marker proteins, Per33-GFP, Rtn1-GFP, and Hmg1-GFP. Similar to the *scs2Δ scs22Δ* strain, the *scs22Δ scs2^K40N/T42A^* mutant displayed impaired ER-phagy (*SI Appendix*, Fig. S8 *A*–*C*), but no substantial defect in bulk autophagy (*SI Appendix*, Fig. S8*D*). These data indicate that the ability of Scs2 to interact with FFAT motif–containing proteins is not only essential for its interaction with Atg40, it is also essential for its function in ER-phagy.

### Scs2 and Atg40 Colocalize at the Edge of ER Sheets or ER Tubules.

**Fig. 6.**
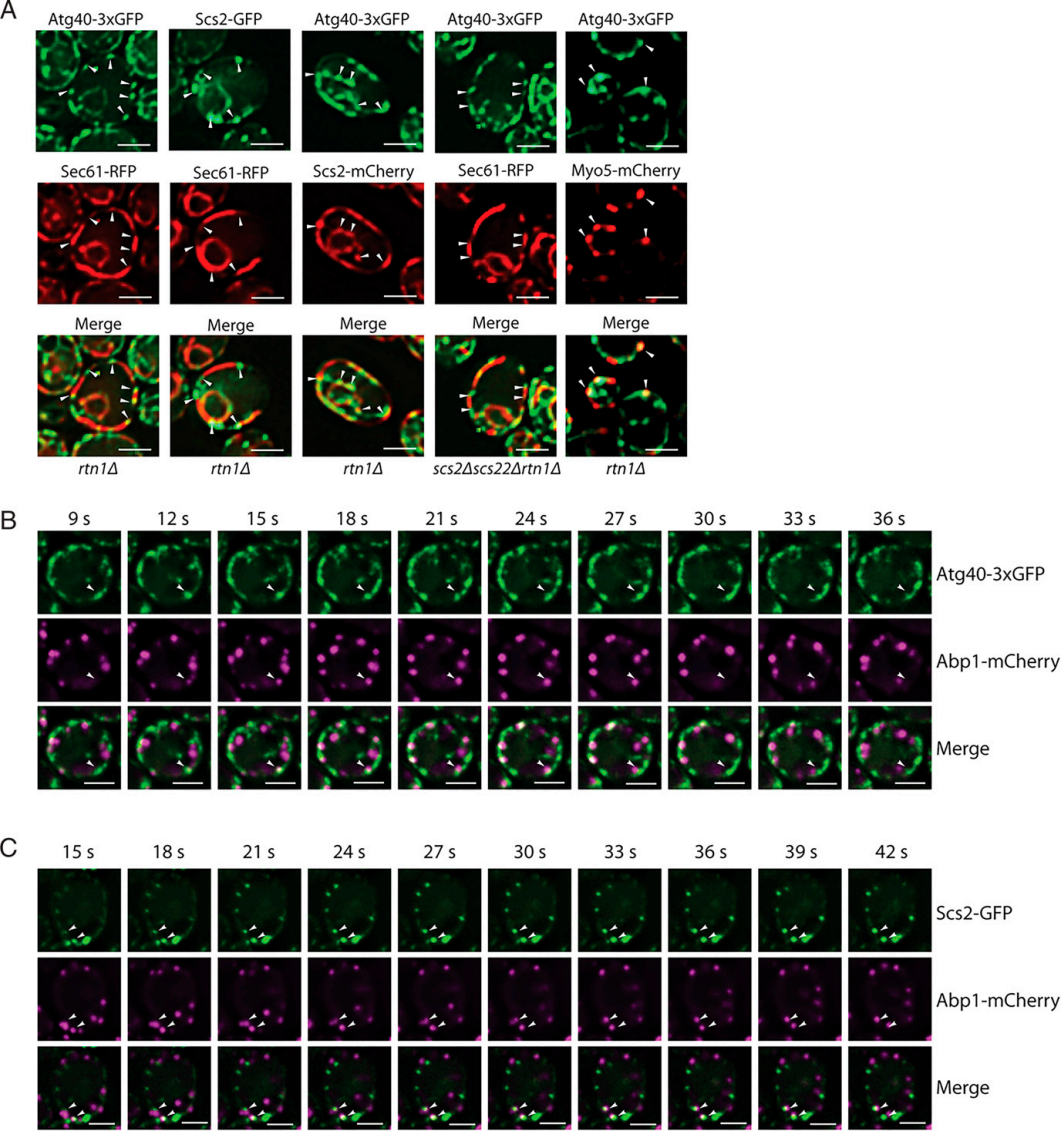
(*A*) Atg40 colocalizes with Scs2 and Myo5 at the edge of ER sheets and tubules. Cells expressing Atg40-3xGFP and Sec61-RFP (first column from *Left*), Scs2-GFP and Sec61-RFP (second column), and Atg40-3xGFP and Scs2-2xmCherry (third column) were incubated with rapamycin for 3 h, collected, and examined by fluorescence microscopy. All strains are in an *rtn1Δ* strain background. Arrowheads point to Atg40 or Scs2 puncta and their corresponding location in the Sec61-RFP channel. (Scale bars, 2 μm.) Cells expressing Atg40-3xGFP and Sec61-RFP in an *scs2Δ scs22 Δrtn1Δ* strain (fourth column) were subjected to rapamycin treatment for 3 h, collected, and examined by fluorescence microscopy. Cells expressing Atg40-3xGFP and Myo5-mCherry in an *rtn1Δ* strain background (fifth column) were subjected to rapamycin treatment for 3 h, collected, and examined by fluorescence microscopy. Arrowheads point to Atg40 puncta and the corresponding location in the red and merged channels. (Scale bars, 2 μm.) (*B* and *C*) Actin-binding protein Abp1 transiently colocalizes with Atg40 and Scs2. (*B*) Cells expressing Atg40-3xGFP and Abp1-mCherry in an *rtn1Δ* strain background were treated with rapamycin for 3 h, collected, and examined by fluorescence microscopy. A 3-min movie with 54 total frames per channel was taken. The frames shown are 9 to 36 s. Arrowheads point to the same location in single and merged channels. (Scale bars, 2 μm.) (*C*) Cells expressing Scs2-GFP and Abp1-mCherry in an *rtn1Δ* strain background were treated with rapamycin for 3 h, collected, and examined by fluorescence microscopy. A 3-min movie with 54 frames per channel was taken. The frames shown are 15 to 42 s. Arrowheads point to the same location in single and merged channels. (Scale bars, 2 μm.)

The physical interaction between Scs2 and Atg40 prompted us to closely examine their localization. This analysis was facilitated by the use of the *rtn1Δ* mutant. In WT cells, the cER has a large number of fenestrations that are below the resolution limit of standard fluorescence microscopy. The loss of the reticulon protein Rtn1, however, causes the fenestrated cER to collapse to a few large continuous sheets, revealing gaps where the PM is not covered by the cER (26). This property of the *rtn1Δ* mutant helps to localize an ER protein relative to the edges of the sheets (35). When we examined the localization of Sec61-RFP in an *rtn1Δ* strain, this GFP fusion protein was observed on long cortical segments that covered a portion of the cell perimeter. The extremities of these segments represent the edge of an ER sheet (Fig. 6*A*). Interestingly, we found that in many cases, Atg40 or Scs2 puncta were located at, or close to, each end of a Sec61 segment (Fig. 6*A*, first and second columns from *Left*). Consistent with the coimmunoprecipitation (Co-IP) data (Fig. 5*B*), Atg40 and Scs2 puncta tended to colocalize with each other (Fig. 6*A*, third column) and this colocalization was independent of rapamycin treatment. The localization of Atg40 puncta to the edges of the sheets did not depend on Scs2 and Scs22 (Fig. 6*A*, fourth column). We also observed partial colocalization of Atg40 with Myo5 (Fig. 6*A*, fifth column). The localization of Atg40 and Scs2 was not highly dynamic; Atg40 moved only locally (Fig. 6*B* and Movie S1), while Scs2 was quite static (Fig. 6*C* and Movie S2). Taken together, these data suggest that Atg40 and Scs2 are enriched at the edges of ER sheets or tubules. When ER-phagy is induced, these cER domains, where Atg40 and Scs2 both reside, are readily accessible to the endocytic machinery. This may facilitate their presentation to Atg11 and other components of the autophagy machinery.

In the above sections, we discussed the importance of actin assembly at endocytic sites to ER-phagy. To further explore this connection, we set out to test the colocalization of Atg40 or Scs2 with Abp1. Abp1 is an actin-binding protein on the cortical actin cytoskeleton (51). It was previously reported that Abp1 displays a transient association with the edges of the cER (35). When we monitored the dynamic colocalization of Abp1 with either Atg40 or Scs2 over a 3-min period (Movies S1 and S2 and Fig. 6 *B* and *C*), Abp1 was much more dynamic than either Atg40 or Scs2. However, we could capture some Abp1 puncta that moved along the cell cortex, overlapped with Atg40 or Scs2 for a short period of time, and then moved toward the cell interior and disappeared. The transient colocalization of the actin-binding protein Abp1 with Atg40 or Scs2 is consistent with the hypothesis that the contacts between cER and endocytic sites promote actin polymerization (35), which in turn helps to present ER domains marked by Atg40 to sites of autophagosome assembly.

## Discussion

Here we address the function of End3 in ER-phagy. Our studies highlight the role of End3, as well as the components that co-operate with End3 at endocytic sites. These include its binding partner Pan1 and the Arp2/3 complex that forms a branched actin network at the endocytic pit, as well as a protein bridge consisting of Myo3/5, Osh2/3, and Scs2/22, which transiently connects the invaginating endocytic pit to the edges of cER sheets (35). Our findings point to a role for these components early in the ER-phagy pathway, prior to the association of the cER cargo receptor Atg40 with the autophagosome machinery assembly scaffold protein Atg11 and prior to the sequestration of ER fragments within autophagosomes. These studies extend earlier observations which showed that blocking actin polymerization with LatA prevents the colocalization of Atg40 with Atg11 (19) and inhibits ER-phagy (6). We now conclude that it is the branched actin network, formed by the Arp2/3 complex at endocytic sites, which is needed for ER-phagy. Importantly, prior studies demonstrated that the concomitant loss of End3 and Pan1 does not block Arp2/3-directed actin assembly per se, but rather uncouples actin assembly from endocytic sites, leading to actin “comets” roaming within the cytosol (29). Thus, Arp2/3 complex–mediated actin assembly is not sufficient for ER-phagy; actin assembly must be coupled to endocytic pits. We cannot exclude the possibility that ER-phagy requires some component whose localization to endocytic pits is dependent upon actin assembly. Nonetheless, the simplest explanation is that actin assembly is directly involved in ER-phagy. Both the cargo receptor Atg40 and the VAP homolog Scs2 are concentrated at the edges of cER sheets that have been shown to associate with these endocytic pits (16, 35). We show that Atg40 can be cross-linked to Scs2 and that the proposed link between the cER and the endocytic pit provided by Scs2/22, Osh2/3, and Myo3/5 is needed for ER-phagy.

In principle, a simple model could explain the role of these components in the early steps of ER-phagy. Through the actions of End3-Pan1 and the Arp2/3 complexes, actin assembly promotes the invagination of endocytic pits (29). The link between an endocytic pit and the rim of a cER sheet, provided by Scs2/22, Osh2/3, and Myo3/5 (35), could serve as a tether to pull a fragment of the cER bearing Atg40 toward the cell interior, where it would encounter Atg11. Through its function as a scaffold protein, Atg11 would then recruit the other components of the Atg machinery and thus promote sequestration of an ER fragment by an autophagosome. While this model is appealing, there are challenges to visualization of these events. ER-phagy is quite slow, requiring hours for significant turnover of ER, whereas endocytosis occurs in minutes. Therefore, one can anticipate that only a minor fraction of endocytic events would be accompanied by the delivery of an ER fragment into the cell interior. This could explain why we do not observe Atg40 puncta in a stable association with Abp1 as the Arp2/3 complex–driven invagination of the endocytic pit progresses (Fig. 6*B*). Another consideration is that Myo3/5, which constitutes an element of the link connecting the endocytic pit to the cER, remains at the base of the pit, not at the invaginating tip (41). Thus, it does not appear to be well situated to help pull a fragment of the cER in from the cortex. Encinar Del Dedo et al. (35) have shown that Scs2/22, Osh2/3, and Myo3/5 are needed to promote actin assembly by the Arp2/3 complex at endocytic sites. Since the Arp2/3 complex function is needed for ER-phagy, this activity could account for the role of these components in ER-phagy. Importantly, loss of Rvs167 blocks endocytosis but not ER-phagy, suggesting a role for actin assembly that is independent of endocytosis.

We found that the FFAT domain–binding function of Scs2 is required for its interaction with Atg40, as well as its role in ER-phagy; however, Atg40 does not contain a convincing FFAT motif, and mutagenesis of the best candidate sites has no effect on the binding to Scs2. This strongly suggests the involvement of a FFAT motif–containing protein that links Scs2 to Atg40. We have ruled out the most obvious candidates, Osh2, Osh3, and Vps13. Interestingly, mammalian cells use VAP proteins to recruit CALCOCO1, a soluble ER-phagy receptor (52), and *Schizosaccharomyces pombe* VAP proteins have been implicated in stress-induced ER turnover, in part through their recruitment of Epr1, a soluble ER-phagy receptor (53). *S. cerevisiae* does not have a clear homolog of Epr1 or CALCOCO1. Recently, a mammalian Scs2 homolog, VAPB, was found to coprecipitate with the ER-phagy receptor RTN3L (15). Further studies will be needed to identify the component that links Atg40 to Scs2 and to assess its role in ER-phagy.

A recent study has shown that AtEH/Pan1, a plant Pan1 homolog, is required for autophagosome assembly at ER–PM contact sites and remains in association with autophagosomes as they mature (54). Similarly, Arp2/3 complex–mediated actin assembly and ER–PM contact sites are needed for autophagosome assembly in mammalian cells (55, 56). We have found no significant effect of the loss of End3-Pan1, Scs2/22, Osh2/3, or Myo5 on bulk autophagy, which is strictly dependent upon autophagosome assembly. The lack of a requirement for these components in autophagosome assembly in yeast has allowed us to reveal a more specific involvement in ER-phagy.

With this study and prior ones, we can begin to propose a possible framework for the early events in ER-phagy. When ER-phagy is induced, the increased expression of Atg40 leads to its concentration at the edges of ER sheets and in ER tubules. These Atg40 concentrations associate with the VAP protein, Scs2, through a FFAT domain–containing protein. A protein bridge consisting of Scs2/22, Osh2/3, and Myo3/5 links these sites to endocytic pits, promoting actin polymerization by the Arp2/3 complex. The localized polymerization of actin not only promotes the invagination of the endocytic pit, leading to the formation of an endocytic vesicle, it also promotes the delivery of ER domains carrying Atg40 to perivacuolar sites where Atg40 interacts with Atg8 and the Lst1-Sec23 complex (13). It will be important to understand the mechanistic details by which actin assembly promotes the delivery of ER to sites of autophagosome assembly.

## Materials and Methods

### Strains and Growth Conditions.

Yeast strains and plasmids used in this study are listed in *SI Appendix*, Tables S1 and S2. All yeast deletion strains and proteins with an AID tag were constructed using the approach of Longtine et al. (57). All other tagged fusion proteins, including those tagged with GFP, mCherry, and RFP, were constructed by using integration or CEN vectors.

ER-phagy was induced as previously described (19). Briefly, yeast cells were grown to an optical density at 600nm (OD_600_) of 0.1 to 0.2 in synthetic complete or dropout medium containing 2% glucose (SD). Rapamycin was added to a final concentration of 200 ng/mL, and the cells were incubated for another 3 to 16 h at 30 °C. For experiments requiring drug treatment, IAA (250 μM final concentration from a stock dissolved in 100% EtOH) or CK-666 (250 μM final concentration from a stock dissolved in 100% DMSO) was added for about 90 min before adding rapamycin.

### Vacuole Staining with fn4-64.

FM4-64 was used to stain the vacuole membrane (58). Rapamycin-treated cells were collected and then resuspended in 100 μL of yeast extract-peptone-dextrose (YPD) media containing 1.6 μM fn4-64. After incubation at 30 °C for 40 to 60 min, cells were pelleted and washed twice with 1 mL YPD media. Cells were finally resuspended in synthetic complete (SC) medium and mounted for examination by an Axio Imager Z1 fluorescence microscope as described below.

To track endocytosis, we used a different staining protocol (59). Cells expressing Myo3-AID1 and Myo5-AID2 were grown to OD_600_ ∼0.5, and either EtOH or IAA was added. After 90 min, about 8 OD_600_ cells were harvested and resuspended in 200 μL of YPD media containing 40 μM fn4-64 and 250 μM IAA or EtOH. For *rvs167Δ* yeast strain, cells were harvested when OD_600_ was ∼0.8 and directly stained with fn4-64. After being incubated on ice for 1 h, cells were quickly rinsed with YPD and then imaged after 1 h (Myo3-AID1 Myo5-AID2) or 2 h (*rvs167Δ*) incubation at room temperature.

### Fluorescence Microscopy.

About 1 OD_600_ cell equivalents were pelleted and imaged on an Axio Imager Z1 fluorescence microscope as described previously (23). This microscope is equipped with an α-Plan apochromat 100×/1.46 ultraviolet-visible infrared objective lens (Zeiss), a Compact Light Source HXP 120V, and an Axiocam 506 mono digital camera. Acquired images were further processed with Open Lab (Improvision), Fiji ImageJ, or Photoshop CS4 (Adobe) software. Movies were taken with a Yokogowa ×1 spinning disk confocal system with a 100× Apo objective and a prime 95B back-thinned complementary metal-oxide semiconductor (CMOS) camera (Teledyne Photometrics).

### Vacuolar GFP Cleavage Assays.

GFP cleavage assays were performed as previously described (23). Approximately 5.0 OD_600_ equivalents of cells expressing Per33-GFP, Rtn1-GFP, Hmg1-GFP, or GFP-Atg8 were harvested by centrifugation at 3,000 × *g* for 5 min. After rinsing with cold water, cells were resuspended in 1 mL of ice-cold 10% trichloroacetic acid (TCA) and kept on ice for at least 30 min. Cells were then pelleted at 13,000 × *g* for 3 min and washed twice with 1 mL of ice-cold acetone to remove residual TCA. Cell pellets were dried in a culture hood and resuspended in 50 μL of resuspension buffer [50 mM Tris⋅HCl, 1 mM ethylenediaminetetraacetic acid (EDTA), 1% sodium dodecyl sulfate (SDS), and 6 M urea, pH 7.5]. About 100 μL of silica beads were added to each sample, and the mixture was then vortexed for 5 min followed by a 5-min incubation at 55 °C in a water bath. Fifty microliters of sample buffer (150 mM Tris⋅HCl, 6% SDS, 6 M urea, 10% 2-mercaptoethanol, and 0.002% bromophenol blue, pH 6.8) was added, and the mixture was vortexed for 5 min and incubated at 55 °C for 10 min in a water bath. All samples were centrifuged at 13,000 × *g* for 10 min to remove debris and subjected to sodium dodecyl sulphate–polyacrylamide gel electrophoresis (SDS-PAGE). All GFP-tagged fusion proteins and free GFP were detected by Western blot using anti-GFP mouse monoclonal antibody (1:2,000 dilution, Roche, clones 7.1 and 13.1). Images were taken and analyzed using Bio-Rad Image Lab software. The cleavage is the amount of free GFP over the total amount of free GFP plus the GFP-tagged protein. The cleavage of each mutant strain was normalized to WT, which was set as 100%.

### Vacuolar Alkaline Phosphatase Activity.

Alkaline phosphatase (ALP) activity assays were performed as described previously (60) with minor modifications. Briefly, cells expressing the cytoplasmic form of Pho8 (Pho8Δ60) were grown in SD dropout medium to OD_600_ ∼0.2. Rapamycin was added to a final concentration of 200 ng/mL to induce autophagy, and cells were then incubated for another 3 or 16 h at 30 °C. About 5 OD_600_ cell equivalents were harvested and washed with 1 mL ice-cold 0.85% NaCl containing 1 mM phenylmethylsulfonyl fluoride (PMSF). Cell pellets were then resuspended in 300 μL of ice-cold lysis buffer [20 mM piperazine-N,N′-bis(2-ethanesulfonic acid) (Pipes), pH 7.0, 0.5% Triton X-100, 50 mM KCl, 100 mM potassium acetate, 10 mM MgSO_4_, 10 μM ZnSO_4_, and 1 mM PMSF] and a half volume (150 μL) of silica beads. The samples were vortexed five times for 1 min at 4 °C, with 1-min intervals on ice. Lysed cells were centrifuged at 14,000 × *g* for 5 min at 4 °C to remove debris. Supernatants were collected, and the protein concentration was measured using the Bradford reagent (Bio-Rad).

To analyze ALP activity, 100 μL 10-times-diluted supernatant was mixed thoroughly with 400 μL of the ALP substrate solution (1.25 mM p-nitrophenyl phosphate, 250 mM Tris⋅HCl, pH 8.5, 0.4% Triton X-100, 10 mM MgSO_4_, and 10 μM ZnSO_4_). The mixture was subsequently incubated at 37 °C for 10 min in a water bath, and the reaction was then terminated by adding 500 μL of stop buffer (1 M glycine/KOH, pH 11.0). The amount of p-nitrophenol was measured at OD_400_. ALP activity was normalized to equal protein concentrations, and the activity for mutants was normalized to the WT.

### Co-IP.

Strains that express Flag-tagged Atg39, Atg40, or their mutants and HA-tagged Scs2 or its mutants were grown at 30 °C to an OD_600_ of ∼0.1. For the strains that require induced expression, CuSO_4_ was added to a final concentration of 125 μM. After a 2-h incubation, rapamycin was added to a final concentration of 200 ng/mL, and the cells were incubated for another 3 h before being harvested. Cells were then pelleted, washed once with water, and resuspended with 1 mL of lysis buffer containing the DSP cross-linker (25 mM Hepes, 150 mM NaCl, 1 mM EDTA, and 2 mM DSP). Cell suspensions were mixed on a Nutator at 4 °C for 2 h. Tris⋅HCl, pH 7.5, 1 M, was added to the above suspensions to a final concentration of 100 mM to stop the cross-linking reaction. Suspensions were incubated on ice for about 15 min, and then protease inhibitor was added. The cell suspensions were then transferred into 2-mL screw cap tubes (Thermo Fisher Scientific) with 2 g zirconia/silica 0.5-mm beads (Biospec Products, Inc.) prewashed with ice-cold lysis buffer without protease inhibitor, and shaken twice for 3 min with a 1-min interval on ice. Triton X-100 was added to a 1% final concentration, and the lysate was incubated at 4 °C for 15 min. The lysate was then spun at 20,000 × *g* for 30 min, and the supernatant was incubated with 10 μL settled anti-Flag beads (Sigma-Aldrich) at 4 °C overnight. Beads were washed five times with lysis buffer containing 0.1% Triton X-100. Bound proteins were released with Laemmli sample buffer and subjected to Western blotting with a rabbit anti-HA (Abcam, 1:2,500) or a rabbit anti-Flag antibody (Sigma-Aldrich, 1:1,000) to detect the tagged proteins. A specific rabbit antiserum was used to detect Ufe1 (Ferro-Novick’s laboratory, 1:1,000).

### Electron Microscopy.

Cells were grown in synthetic media before inducing autophagy by addition of 200 ng/mL rapamycin for 12 h. Electron microscopy was performed as previously described (13). Briefly, cells were fixed in potassium permanganate and embedded in Spurr’s resin. After polymerization, 55- to 60-nm thin sections were cut using an Ultracut ultramicrotome (Leica Microsystems), transferred onto formvar carbon-coated copper grids, and stained before viewing. The percentage of autophagic bodies (ABs) containing ER fragments was statistically assessed by counting 500 randomly selected AB profiles over three grids for each analyzed condition. In our electron microscopy preparations, the cER appeared as well-defined electron-dense (dark) tubules with a width of 3 to 5 nm. These tubules were distributed along the PM and were sometimes distinctively connected to the nuclear envelope. ER fragments within ABs had a length of at least 25 nm and were identified based on this morphology.

## Supplementary Material

Supplementary File

Supplementary File

Supplementary File

## Data Availability

All study data are included in the article and/or supporting information.
