# Supplementary material for: ER-phagy requires the assembly of actin at sites of contact between the cortical ER and endocytic pits
Source: Proc Natl Acad Sci U S A. 2022 Jan 31;119(6):e2117554119. doi: 10.1073/pnas.2117554119 (PMC8833162; doi:10.1073/pnas.2117554119)
Supplement: Supplementary File [file pnas.2117554119.sapp.pdf]

PNAS
www.pnas.org

Supplementary Information for

ER-phagy requires the assembly of actin at sites of contact between the cortical ER and endocytic pits

Dongmei Liu [1], Muriel Mari [2], Xia Li[1], Fulvio Reggiori [2], Susan Ferro-Novick [1] and Peter Novick [1]

Peter Novick and Susan Ferro-Novick
Email: pnovick@health.ucsd.edu or sferronovick@health.ucsd.edu

This PDF file includes:

Figures S1 to S8
Tables S1 to S2
Legends for Movies S1 to S2

Other supplementary materials for this manuscript include the following:

Movies S1 to S2

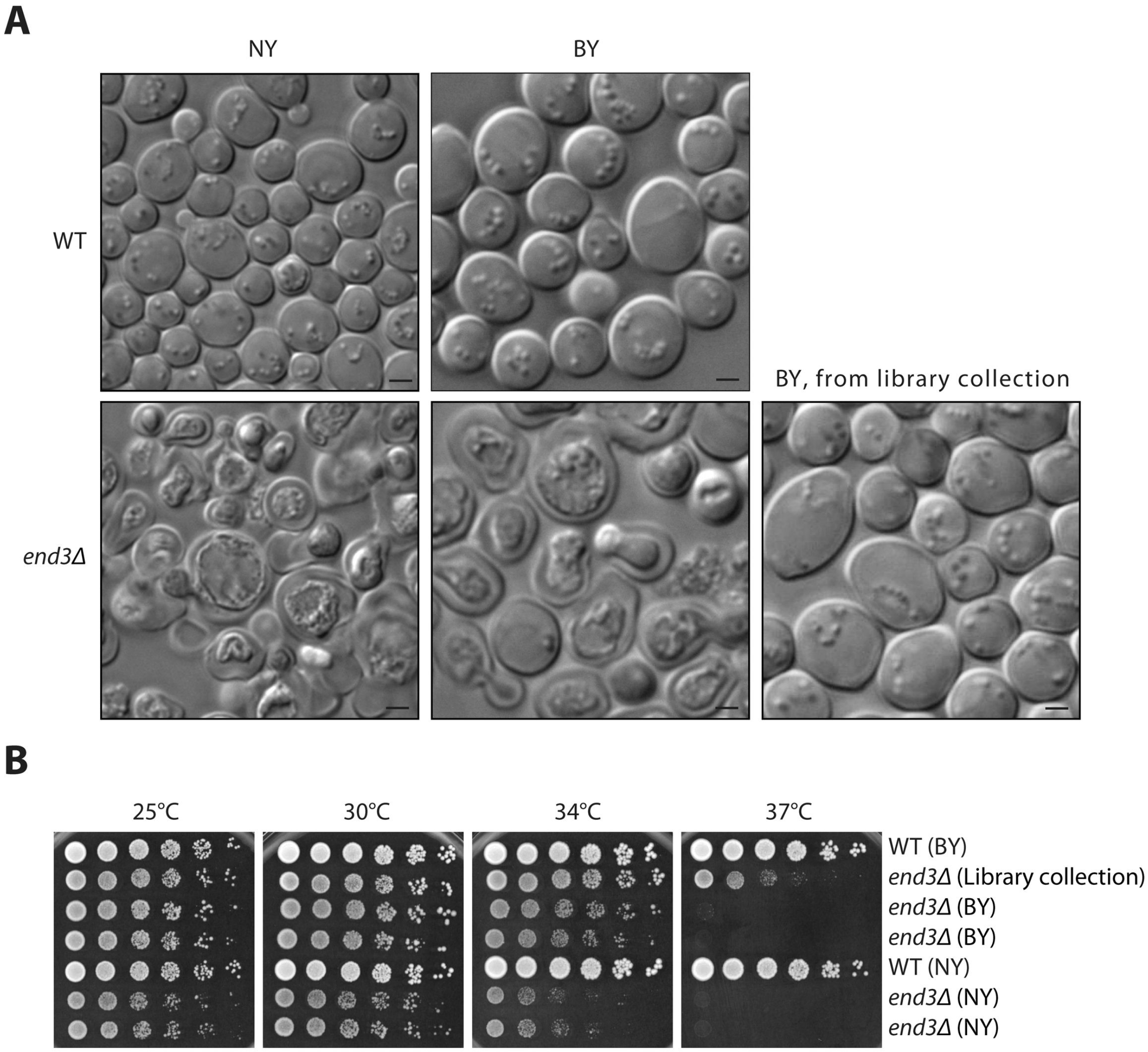

Fig. S1. The *end3Δ* strain from the deletion library collection displays altered morphology and growth. (A) Rtn1 was tagged with GFP in WT (NY background), *end3Δ* (NY background), WT (BY background), *end3Δ* (BY background) and *end3Δ* (BY background from deletion library collection). Cells were induced with rapamycin for 16 h and then a DIC image was acquired. Scale bar, 2 μm. (B) Indicated strains were grown to an early log phase in YPD medium at 25°C, serially diluted and spotted on YPD plates. The plates were then incubated for 2 d at 25°C, 30°C, 34°C and 37°C, respectively.

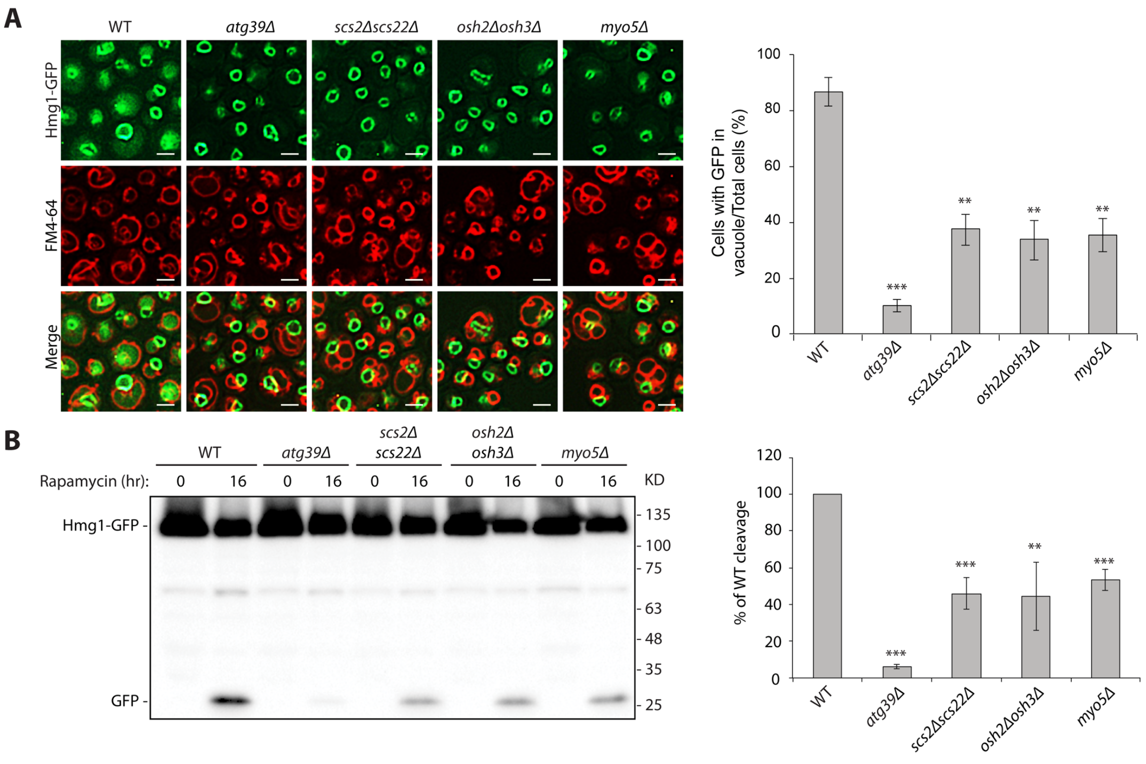

Fig. S2. Proteins linking the cER to endocytic sites are important for delivery of Hmg1 into the vacuole. (A) Scs2/Scs22, Osh2/Osh3 and Myo5 are required for delivery of Hmg1 into the vacuole. Left panel, fluorescence images of cells expressing Hmg1-GFP. Cells were treated with rapamycin for 16 h and vacuoles were stained with FM4-64. Right panel, the percentage of cells with GFP in the vacuole was quantified. Error bars represent SD, N = 3 independent experiments. **, $P < 0.01$; ***, $P < 0.001$. Student's t-test. Scale bar, 2 μm. (B) Left panel, western blot of Hmg1-GFP cleavage after cells were treated with rapamycin for 16 h. Right panel, percentage of free GFP divided by the total GFP amount was quantified. WT was set as 100%. All mutants were normalized to the WT control. Error bars represent SD, N = 3 independent experiments. **, $P < 0.01$; ***, $P < 0.001$. Student's t-test.

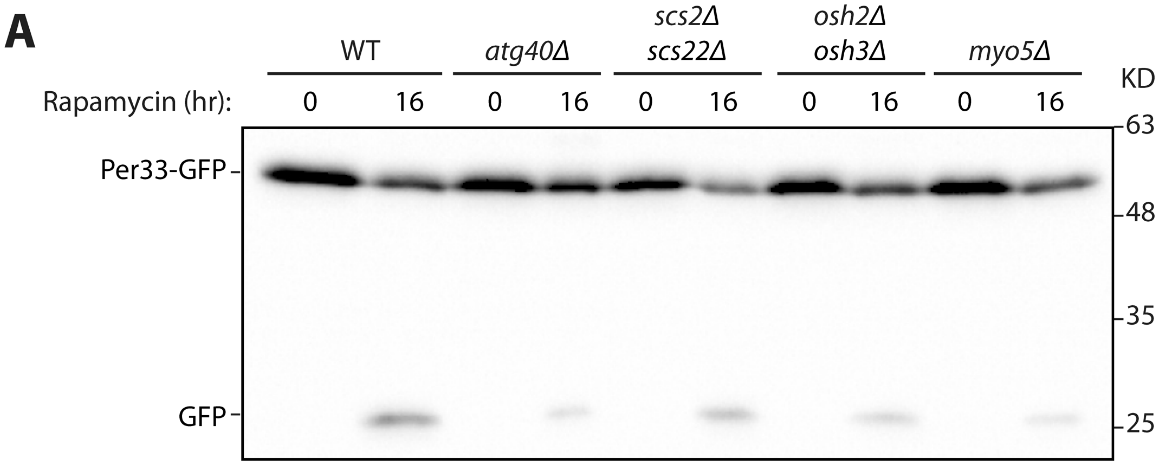

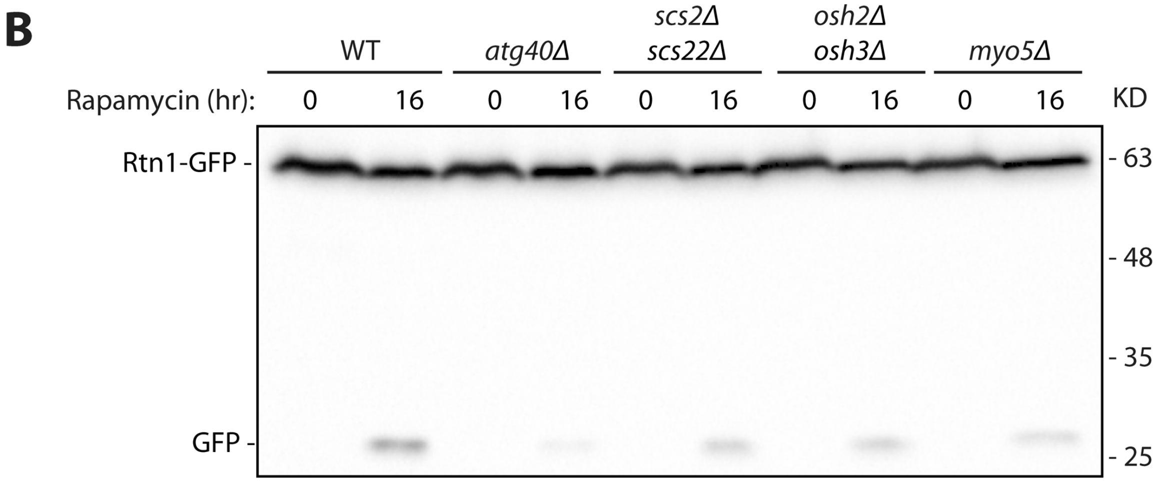

Fig. S3. Proteins linking the cER to endocytic sites are important for ER-phagy. (A) Western blot analysis of Per33-GFP cleavage after cells were treated with rapamycin for 16 h. This is the same blot as Fig. 2B, but with a shorter exposure time. (B) Western blot analysis of Rtn1-GFP cleavage after cells were treated with rapamycin for 16 h. This is the same blot as Fig. 2D, but with a shorter exposure time.

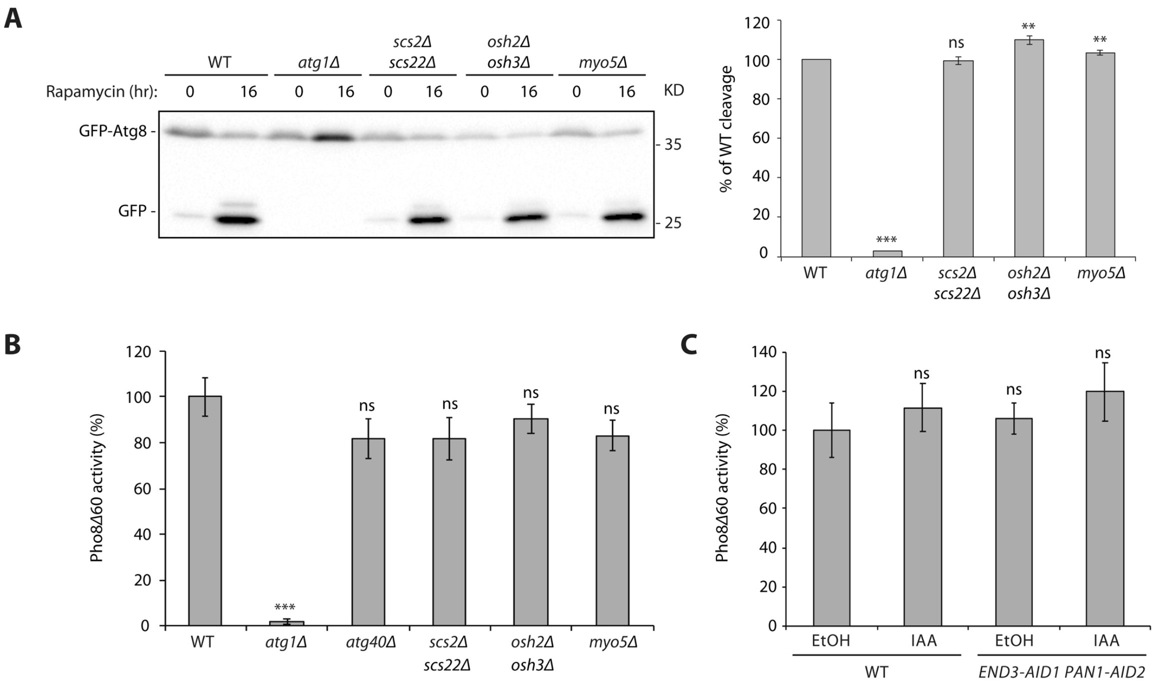

Fig. S4. Scs2/Scs22, Osh2/Osh3 and Myo5 are not involved in bulk autophagy. (A) Left panel. Western blot of GFP-Atg8 cleavage after cell treatment with rapamycin for 16 h. Right panel. The percentage of free GFP divided by the total GFP amount was quantified. WT was set at 100% and all mutants were normalized to the WT control. Error bars represent SD, N = 3 independent experiments. NS, non-significant ($P \geq 0.05$); **, $P < 0.01$. ***, $P < 0.001$. Student's t-test. (B) ALP activity was analyzed in cells expressing Pho8Δ60 as described in the Materials and Methods. Autophagy was induced with rapamycin for 16 h. WT was set at 100% and all mutants were normalized to the WT control. Error bars represent SD, N = 3 independent experiments. NS, non-significant ($P \geq 0.05$). ***, $P < 0.001$. Student's t-test. (C) ALP activity was analyzed in WT and the End3-AID1- and Pan1-AID2-co-expressing strain that was treated with either EtOH or IAA before incubation with rapamycin for 3 h. EtOH-treated WT cells were set at 100% and others were normalized to the EtOH-treated WT cells. Error bars represent SD, N = 3 independent experiments. NS, non-significant ($P \geq 0.05$). Student's t-test.

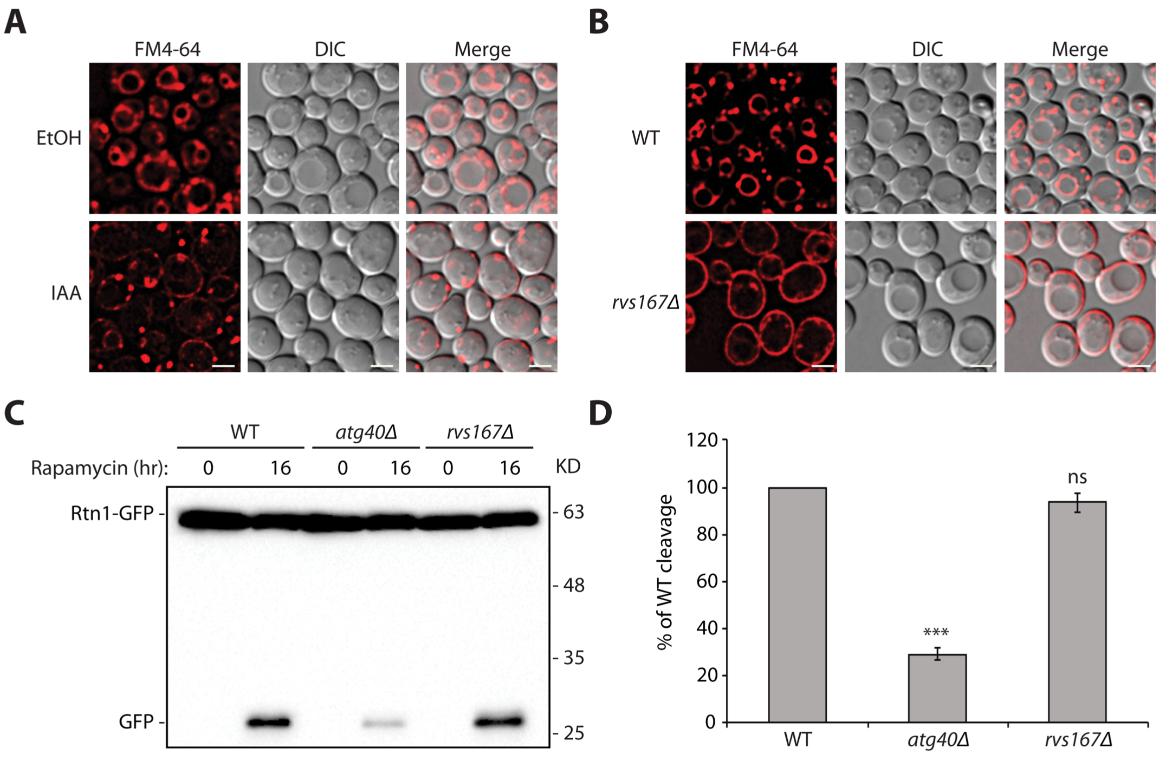

Fig. S5. (A) Induced degradation of Myo3 and Myo5 blocks endocytic uptake of FM4-64. Cells expressing *MYO3-AID1 and MYO5-AID2* were pre-treated with either EtOH or IAA for 90 min before the addition of FM4-64. Uptake was assayed as described in the Materials and Methods. **(B-D) Deletion of *rvs167* blocks endocytosis but not ER-phagy** (B). Uptake of FM4-64 was assayed as described in the Materials and Methods. (C) The *rvs167Δ* strain is not deficient in cER-phagy. Western blot analysis of Rtn1-GFP cleavage after WT, *atg40* and *rvs167Δ* cells were treated with rapamycin for 16 h. (D) The percentage of free GFP divided by the total GFP in (C) was quantified. WT was set as 100%. All mutants were normalized to the WT control. Error bars represent SD, N = 3 independent experiments. ***, $P < 0.001$. NS, non-significant ($P \geq 0.05$). Student's t-test.

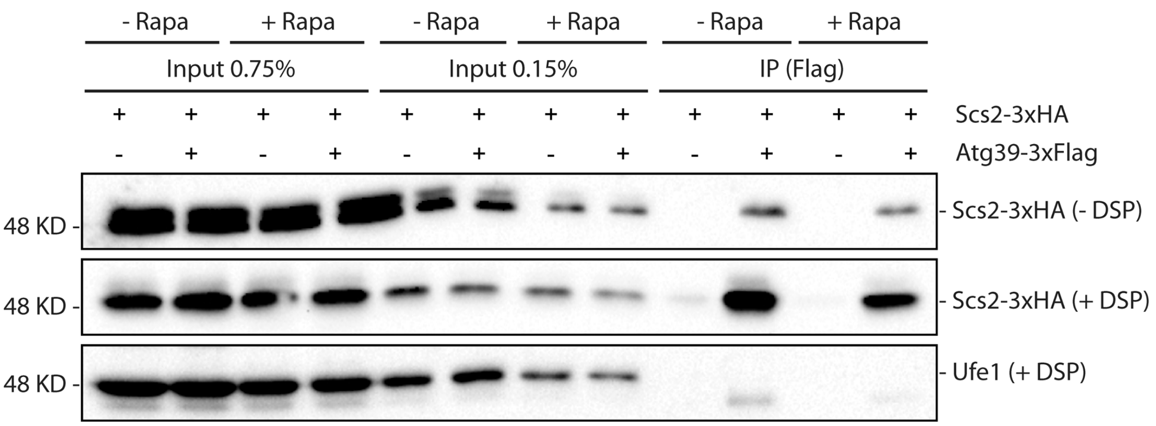

Fig. S6. Scs2 interacts with Atg39 *in vivo*. Atg39-3xFlag was precipitated from lysates of cells treated with or without rapamycin. Cell lysates were pretreated with (+ DSP) or without (-DSP) the crosslinking reagent DSP. The precipitates were blotted with anti-HA antibodies to detect Scs2-3xHA, and the Ufe1 rabbit antiserum to detect Ufe1.

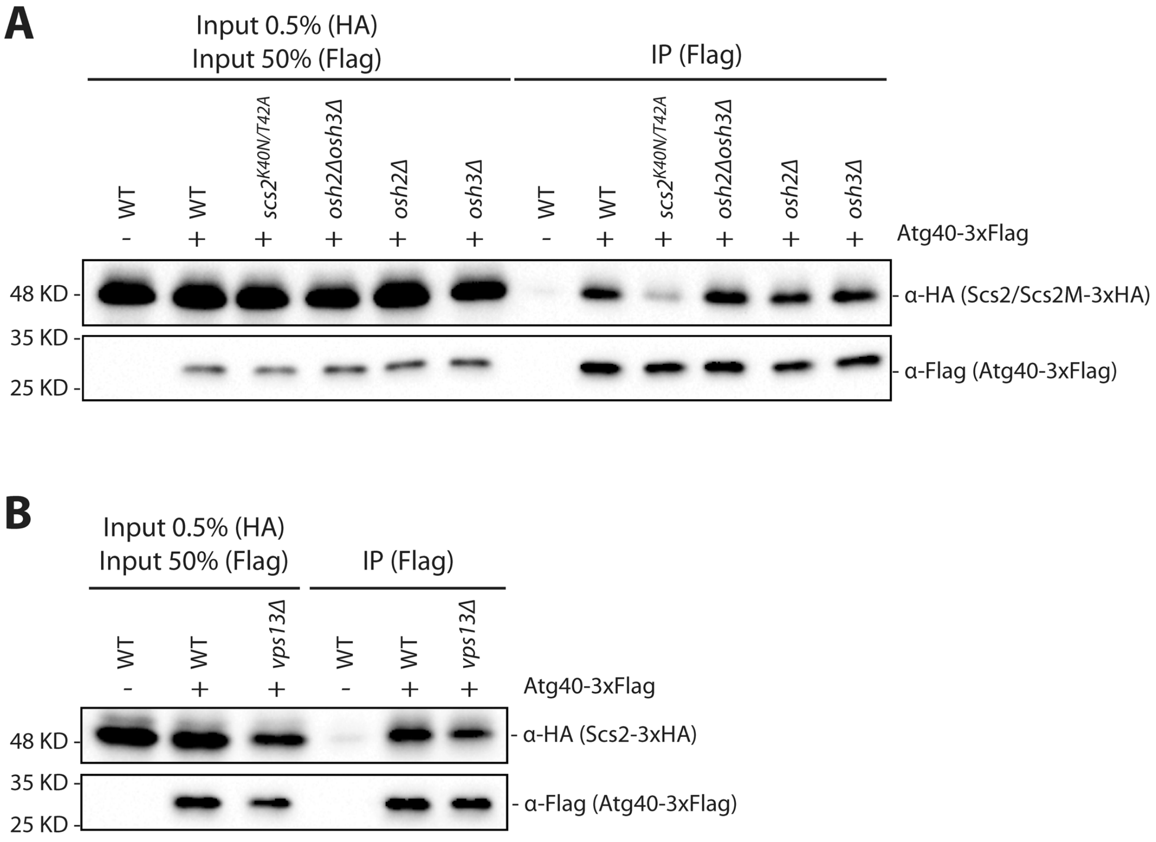

Fig. S7. Osh2, Osh3 and Vps13 are not needed for the Atg40-Scs2 interaction. Atg40-3xFlag was immunoprecipitated from WT and several *osh∆* mutants (A) or a *vps13∆* mutant (B), as indicated. The precipitates were blotted with anti-HA antibodies to detect Scs2-3xHA, and anti-Flag antibodies to detect Atg40-3xFlag. Crosslinking reagent DSP was used prior to cell lysis.

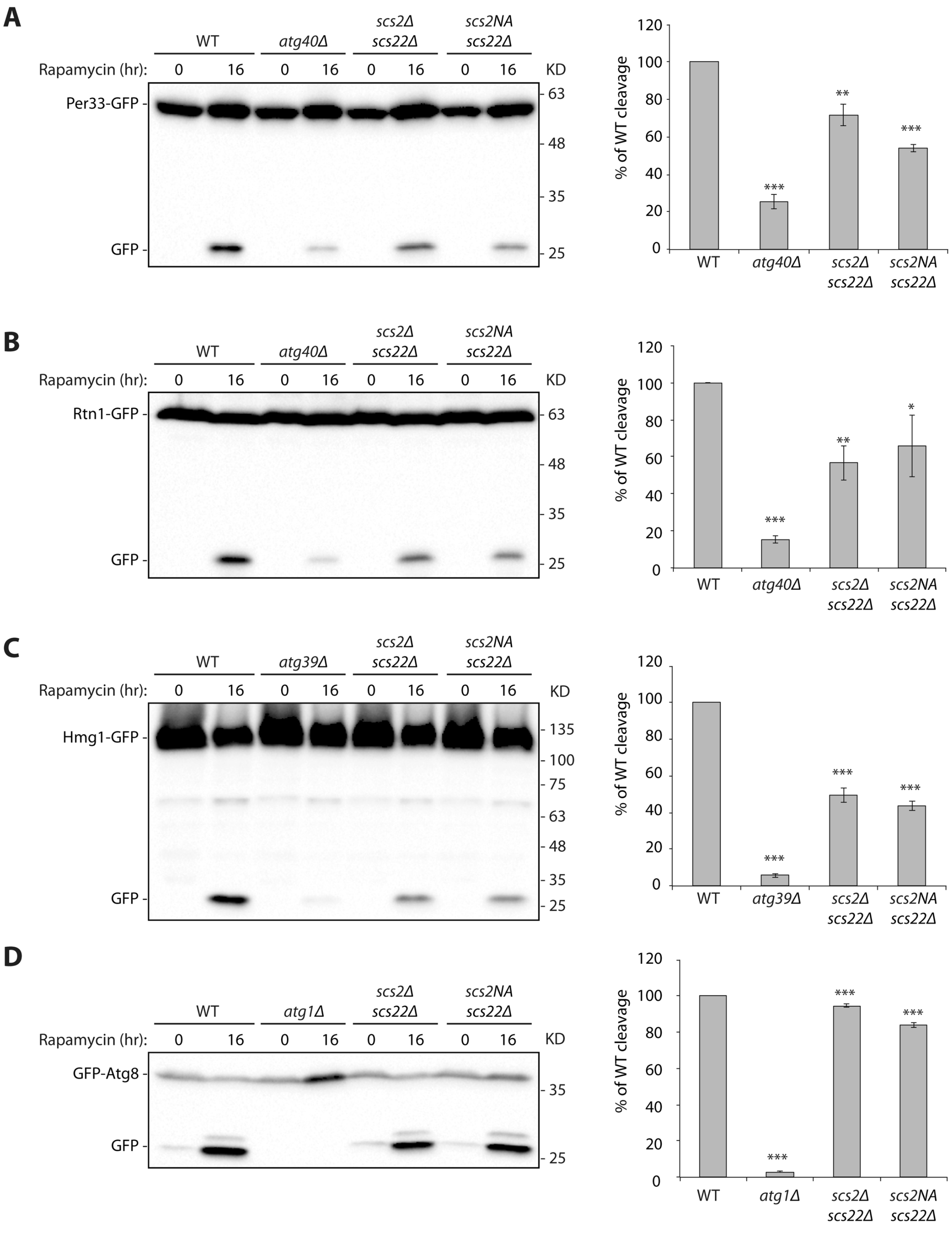

Fig. S8. The FFAT domain binding motif of Scs2 is important for its function in ER-phagy. Left panel. Western blot of cells after treatment with rapamycin for 16 h. The anti-GFP antibody was used to detect free GFP and GFP fusion proteins, Per33-GFP (A), Rtn1-GFP (B), Hmg1-GFP (C), and GFP-Atg8 (D). Right panel. The percentage of free GFP divided by the total GFP

amount was quantified. WT was set as 100%. All mutants were normalized to the WT control. Error bars represent SD, N = 3 independent experiments. *, $P < 0.05$; **, $P < 0.01$; ***, $P < 0.001$. Student's t-test.

Table S1. Plasmids for yeast transformation

NRB	Plasmid name	Source
SFNB2206	pRS315-Rtn1-GFP (CEN, LEU2)	Ferro-Novick lab collection
SFNB2288	pRS306-Atg40ΔN-3xEGFP (URA3, BstEII digestion for integration into *ATG40* locus)	Ferro-Novick lab collection
NRB1660	pRS303-Atg11-2xmCherry (HIS3, EcoRV digestion to integrate into *ATG11* locus)	This study
SFNB2335	pRS305-Per33-GFP (LEU2, BmgBI digestion to integrate into *PER33* locus)	Ferro-Novick lab collection
SFNB2374	pRS305-Hmg1ΔN-GFP (LEU2, XbaI digestion for integration into *HMG1* locus)	Ferro-Novick lab collection
SFNB1637	pRS416-GFP-Atg8 (CEN, URA3)	Ferro-Novick lab collection
NRB1661	pRS305-Scs2-3xHA (LEU2, PstI digestion for integration into *SCS2* locus)	This study
NRB1662	pRS426-pCUP1-ATG40-3xFLAG (2 μ, URA3)	This study
NRB1663	pRS305-pScs2-Scs2K40N/T42A-3xHA (LEU2, HindIII digestion for integration into *SCS2* promoter locus)	This study
NRB1664	pRS426-pCUP1-Atg40F107A/E112A-3xFLAG (2 μ, URA3)	This study
NRB1665	pRS426-pCUP1-Atg40F233A/E237A-3xFLAG (2 μ, URA3)	This study
NRB1666	pRS426-pCUP1-Atg40F34A-3xFLAG (2 μ, URA3)	This study
NRB1667	pRS306-pScs2-Scs2K40N/T42A-3xHA (URA3, HindIII digestion for integration into *SCS2* promoter locus)	This study
SFNB2201	pRS305-sec61ΔN-2xmRFP	Ferro-Novick lab collection
NRB1668	pRS305-Scs2-GFP (LEU2, BstAPI digestion for integration into *SCS2* locus)	This study
NRB1669	pRS306-Scs2-2xmCherry (URA3, BstAPI digestion for integration into *SCS2* locus)	This study
NRB1670	pRS306-Abp1-mCherry (URA3, XbaI digestion for integration into *ABP1* locus)	This study
NRB1671	pRS305-Myo5-3xHA (LEU2, BglII digestion for integration into *Myo5* locus)	This study

Table S2. Yeast strains used in this study

NY	Yeast genotype	Source
NY3323	*MATa, leu2-3, 112, his3Δ200, ura3-52, Gal+ [end3Δ::KanMX6]*	This study
NY3324	*MATa, leu2-3, 112, his3Δ200, ura3-52, Gal+ [end3Δ::KanMX6] [RTN1-GFP::LEU2, CEN]*	This study
SFNY2088	*MATa, ura3-52, leu2-3,112, his3Δ200, [RTN1-GFP::LEU2, CEN]*	Ferro-Novick lab collection
NY3325	*BY4741 MATa his3Δ1 leu2Δ0 met15Δ0 ura3Δ0 [end3Δ::KanMX6]*	This study
NY3326	*BY4741 MATa his3Δ1 leu2Δ0 met15Δ0 ura3Δ0 [end3Δ::KanMX6] [RTN1-GFP::LEU2, CEN]*	This study
SFNY3629	*BY4741 MATa his3Δ1 leu2Δ0 met15Δ0 ura3Δ0 [RTN1-GFP::LEU2, CEN]*	Ferro-Novick lab collection
NY3327	*leu2-3, 112, his3Δ200, ura3-52, [END3-AID1::hphNT] [PAN1-AID2::KanMX6] [TIR1::LEU2] [ATG40-3xGFP::URA3] [ATG11-2xmCherry::HIS3]*	This study
SFNY2962	*MATa, Gal+, ura3-52, leu2-3,112, his3Δ200, [ATG40-3xGFP::URA3] [ATG11-2xmCherry::LEU2]*	Ferro-Novick lab collection
SFNY3113	*MATa, leu2-3, 112, his3Δ200, ura3-52, Gal+ [PER33-GFP::LEU2]*	Ferro-Novick lab collection
SFNY3115	*MATa, leu2-3, 112, his3Δ200, ura3-52, Gal+ [atg40Δ::KanMX6] [PER33-GFP::LEU2]*	Ferro-Novick lab collection
NY3328	*MATa, leu2-3, 112, his3Δ200, ura3-52, Gal+ [scs2Δ::KanMX6] [scs22Δ::HisMX6] [PER33-GFP::LEU2]*	This study
NY3329	*MATa, leu2-3, 112, his3Δ200, ura3-52, Gal+ [osh2Δ::KanMX6] [osh3Δ::HisMX6] [PER33-GFP::LEU2]*	This study
NY3330	*MATalpha, leu2-3, 112, his3Δ200, ura3-52, Gal+ [myo5Δ::HisMX6] [PER33-GFP::LEU2]*	This study
SFNY3139	*MATa, Gal+, ura3-52, leu2-3,112, his3Δ200, [atg40Δ::KanMX6] [RTN1-GFP::LEU2, CEN]*	Ferro-Novick lab collection
NY3331	*MATa, leu2-3, 112, his3Δ200, ura3-52, Gal+ [scs2Δ::KanMX6] [scs22Δ::HisMX6] [RTN1-GFP::LEU2, CEN]*	This study
NY3332	*MATa, leu2-3, 112, his3Δ200, ura3-52, Gal+ [osh2Δ::KanMX6] [osh3Δ::HisMX6] [RTN1-GFP::LEU2, CEN]*	This study
NY3333	*MATalpha, leu2-3, 112, his3Δ200, ura3-52, Gal+ [myo5Δ::HisMX6] [RTN1-GFP::LEU2, CEN]*	This study
NY3334	*MATa, leu2-3, 112, his3Δ200, ura3-52, Gal+ [HMG1-GFP::LEU2]*	This study
SFNY3222	*MATalpha. Gal+, ura3-52, leu2-3,112, his3Δ200, [atg39Δ::His3MX6] [Hmg1-GFP::LEU2]*	Ferro-Novick lab collection
NY3335	*MATa, leu2-3, 112, his3Δ200, ura3-52, Gal+ [scs2Δ::KanMX6] [scs22Δ::HisMX6] [HMG1-GFP::LEU2]*	This study
NY3336	*MATa, leu2-3, 112, his3Δ200, ura3-52, Gal+ [osh2Δ::KanMX6] [osh3Δ::HisMX6] [HMG1-GFP::LEU2]*	This study
NY3337	*MATalpha, leu2-3, 112, his3Δ200, ura3-52, Gal+ [myo5Δ::HisMX6] [HMG1-GFP::LEU2]*	This study
SFNY2860	*MATa ura3-52 leu2-3, 112 his3Δ200 [GFP-ATG8::URA3, CEN]*	Ferro-Novick lab collection
SFNY2861	*MATalpha ura3-52 leu2-3, 112 his3Δ200 [atg1Δ::His3MX6] [GFP-ATG8::URA3, CEN]*	Ferro-Novick lab collection
NY3338	*MATa, leu2-3, 112, his3Δ200, ura3-52, Gal+ [scs2Δ::KanMX6] [scs22Δ::HisMX6] [GFP-ATG8::URA3, CEN]*	This study

NY3339	*MATa, leu2-3, 112, his3Δ200, ura3-52, Gal+ [osh2Δ::KanMX6] [osh3Δ::HisMX6] [GFP-ATG8::URA3, CEN]*	This study
NY3340	*MATalpha, leu2-3, 112, his3Δ200, ura3-52, Gal+ [myo5Δ::HisMX6] [GFP-ATG8::URA3, CEN]*	This study
SFNY2566	*MATalpha his3-200 leu2-3,112 ura3-52 [pho8Δ60::URA3]*	Ferro-Novick lab collection
SFNY2979	*MAT alpha, Gal+, ura3-52, leu2-3,112, his3Δ200, [atg1Δ::His3MX6] [pho8Δ60::URA3]*	Ferro-Novick lab collection
NY3341	*MATa, leu2-3, 112, his3Δ200, ura3-52, Gal+ [scs2Δ::KanMX6] [scs22Δ::HisMX6] [pho8Δ60::URA3]*	This study
NY3342	*MATa, leu2-3, 112, his3Δ200, ura3-52, Gal+ [osh2Δ::KanMX6] [osh3Δ::HisMX6] [pho8Δ60::URA3]*	This study
NY3343	*MATalpha, leu2-3, 112, his3Δ200, ura3-52, Gal+ [myo5Δ::HisMX6] [pho8Δ60::URA3]*	This study
NY3344	*MATalpha Gal+, ura3-52, leu2-3,112, his3Δ200 [pho8Δ60::URA3] [end3Δ::KanMX6]*	This study
NY3345	*MATa Gal+, ura3-52, leu2-3,112, his3Δ200, [pho8Δ60::URA3] [END3-AID1::hphNT] [PAN1-AID2::KanMX6] [TIR1::LEU2]*	This study
NY3346	*MATa, leu2-3, 112, his3Δ200, ura3-52, [MYO3-AID1::hphNT] [MYO5-AID2::KanMX6] [pGPD-TIR1::LEU2] [ATG40-3xGFP::URA3] [ATG11-2xmCherry::HIS3]*	This study
NY3347	*MATa his3Δ1 leu2Δ0 met15Δ0 ura3Δ0 [rvs167Δ::KanMX4] [RTN1-GFP::LEU2]*	This study
SFNY3323	*MATalpha, Gal+, ura3-52, leu2-3,112, his3Δ200, [pep4Δ::KanMX6]*	Ferro-Novick lab collection
SFNY3549	*MAT alpha, Gal+, ura3-52, leu2-3,112, his3Δ200, [pep4Δ::KanMX6] [atg14Δ::His3MX6]*	Ferro-Novick lab collection
SFNY3324	*MATa, Gal+, ura3-52, leu2-3,112, his3Δ200, [pep4Δ::His3MX6] [atg40Δ::KanMX6]*	Ferro-Novick lab collection
NY3348	*MATa ura3-52, leu2-3,112, his3Δ200, [pep4Δ::His3MX6] [scs2Δ::KanMX6] [scs22Δ::His3MX6]*	This study
NY3349	*MATalpha, ura3-52, leu2-3,112, his3Δ200, [pep4Δ::His3MX6] [osh2Δ::KanMX6] [osh3Δ::His3MX6]*	This study
NY3350	*MATa ura3-52 leu2-3, 112 his3Δ200 [SCS2-3xHA::LEU2]*	This study
NY3351	*MATa ura3-52 leu2-3, 112 his3Δ200 [SCS2-3xHA::LEU2] [pRS426-pCUP1-ATG40-3xFLAG::URA3, 2u]*	This study
NY3352	*MATa, leu2-3, 112, his3Δ200, ura3-52, Gal+ [MYO5-3xHA::LEU2]*	This study
NY3353	*MATa, leu2-3, 112, his3Δ200, ura3-52, Gal+ [MYO5-3xHA::LEU2] [pRS426-pCUP1-ATG40-3xFLAG::URA3, 2u]*	This study
NY3354	*MATa, ura3-52, leu2-3,112, his3Δ200, [scs2Δ::KanMX6] [scs2K40N/T42A-3xHA::LEU2] [pCUP1-ATG40-3xFLAG::URA3, 2u]*	This study
NY3355	*MATa, ura3-52, leu2-3,112, his3Δ200, [scs2-3xHA::LEU2] [pCUP1-atg40F107A/E112A-3xFLAG::URA3, 2u]*	This study
NY3356	*MATa ura3-52 leu2-3, 112 his3Δ200 [SCS2-3xHA::LEU2] [pCUP1-atg40F233A/E237A-3xFLAG::URA3]*	This study
NY3357	*MATa ura3-52 leu2-3, 112 his3Δ200 [SCS2-3xHA::LEU2] [pCUP1-atg40F34A-3xFLAG::URA3]*	This study
NY3358	*MATa ura3-52 leu2-3, 112 his3Δ200 [SCS2-3xHA::LEU2] [pRS426-pCUP1-ATG39-3xFLAG::URA3, 2u]*	This study
NY3359	*MATa, leu2-3, 112, his3Δ200, ura3-52, Gal+ [osh2Δ::KanMX6] [SCS2-3xHA::LEU2] [pRS426-pCUP1-ATG40-3xFLAG::URA3, 2u]*	This study
NY3360	*MATalpha, leu2-3, 112, his3Δ200, ura3-52, Gal+ [osh3Δ::HisMX6] [SCS2-3xHA::LEU2] [pRS426-pCUP1-ATG40-3xFLAG::URA3, 2u]*	This study

NY3361	*MATalpha, leu2-3, 112, his3Δ200, ura3-52, Gal+ [osh2Δ::KanMX6] [osh3Δ::HisMX6] [SCS2-3xHA::LEU2] [pRS426-pCUP1-ATG40-3xFLAG::URA3, 2u]*	This study
NY3362	*MATa, ura3-52, leu2-3,112, his3Δ200, [vps13Δ::KanMX6] [SCS2-3xHA::LEU2] [pRS426-pCUP1-ATG40-3xFLAG::URA3, 2u]*	This study
NY3363	*MATa, leu2-3, 112, his3Δ200, ura3-52, Gal+ [scs2Δ::KanMX6] [scs22Δ::HisMX6] [scs2K40N/T42A-3xHA::URA3] [PER33-GFP::LEU2]*	This study
NY3364	*MATa, leu2-3, 112, his3Δ200, ura3-52, Gal+ [scs2Δ::KanMX6] [scs22Δ::HisMX6] [scs2K40N/T42A-3xHA::LEU2] [RTN1-GFP::URA3, CEN]*	This study
NY3365	*MATa, leu2-3, 112, his3Δ200, ura3-52, Gal+ [scs2Δ::KanMX6] [scs22Δ::HisMX6] [scs2K40N/T42A-3xHA::URA3] [HMG1-GFP::LEU2]*	This study
NY3366	*MATa, leu2-3, 112, his3Δ200, ura3-52, Gal+ [scs2Δ::KanMX6] [scs22Δ::HisMX6] [scs2K40N/T42A-3xHA::LEU2] [GFP-ATG8::URA3, CEN]*	This study
NY3367	*MATa, ura3-52, leu2-3,112, his3Δ200, [rtn1Δ::KanMX4] [ATG40-3xGFP::LEU2] [SEC61-2xRFP::URA3]*	This study
NY3368	*MATa, ura3-52, leu2-3,112, his3Δ200, [rtn1Δ::KanMX4] [SCS2-GFP::LEU2] [SEC61-2xRFP::URA3]*	This study
NY3369	*MATa, ura3-52, leu2-3,112, his3Δ200, [rtn1Δ::KanMX4] [ATG40-3xGFP::LEU2] [SCS2-2xmCherry::URA3]*	This study
NY3370	*MATa, leu2-3, 112, his3Δ200, ura3-52, Gal+ [rtn1Δ::KanMX4] [scs2Δ::KanMX6] [scs22Δ::HisMX6] [ATG40-3xGFP::LEU2] [SEC61-2xRFP::URA3]*	This study
NY3371	*MATa, ura3-52, leu2-3,112, his3Δ200, [rtn1Δ::KanMX4] [ATG40-3xGFP::LEU2] [MYO5-mCherry::URA3]*	This study
NY3372	*MATa, ura3-52, leu2-3,112, his3Δ200, [rtn1Δ::KanMX4] [ATG40-3xGFP::LEU2] [ABP1-mCherry::URA3]*	This study
NY3373	*MATa, ura3-52, leu2-3,112, his3Δ200, [rtn1Δ::KanMX4] [SCS2-GFP::LEU2] [ABP1-mCherry::URA3]*	This study

Movie S1 (separate file). Actin-binding protein Abp1 transiently co-localizes with Atg40. Cells expressing Atg40-3xGFP and Abp1-mCherry in an *rtn1Δ* strain background were treated with rapamycin for 3 h, collected and examined by fluorescence microscopy. A 3 min movie with 54 total frames per channel was taken. The final movie is 2 fps.

Movie S2 (separate file). Actin-binding protein Abp1 transiently co-localizes with Scs2. Cells expressing Scs2-GFP and Abp1-mCherry in an *rtn1Δ* strain background were treated with rapamycin for 3 h, collected and examined by fluorescence microscopy. A 3 min movie with 54 total frames per channel was taken. The final movie is 2 fps.
